# Primary HIV prevention in pregnant and lactating Ugandan women: A randomized trial

**DOI:** 10.1371/journal.pone.0212119

**Published:** 2019-02-25

**Authors:** Jaco Homsy, Rachel King, Femke Bannink, Zikulah Namukwaya, Eric Vittinghof, Alexander Amone, Francis Ojok, Gordon Rukundo, Sharon Amama, Juliane Etima, Joyce Matovu, Fitti Weissglas, Lawrence Ojom, Pamela Atim, Lynae Darbes, Josaphat Byamugisha, George Rutherford, Elly Katabira, Mary Glenn Fowler, the PRIMAL Study Team

**Affiliations:** 1 Institute for Global Health Sciences, University of California, San Francisco, CA, United States of America; 2 AVSI Foundation, Kampala, Uganda; 3 Makerere University—Johns Hopkins University Research Collaboration, Kampala, Uganda; 4 Department of Epidemiology and Biostatistics, University of California, San Francisco, CA, United States of America; 5 St Joseph’s Hospital, Kitgum, Uganda; 6 Department of Health Behavior and Biological Sciences, Center for Sexuality and Health Disparities, University of Michigan School of Nursing, Ann Arbor, MI, United States of America; 7 Department of Obstetrics and Gynecology, Makerere University School of Medicine, Kampala, Uganda; 8 Department of Medicine & College of Health Sciences, Makerere University School of Medicine, Kampala, Uganda; 9 Department of Medicine, Johns Hopkins University, Baltimore, MD, United States of America; International AIDS Vaccine Initiative, UNITED STATES

## Abstract

**Background:**

The ‘Primary HIV Prevention among Pregnant and Lactating Ugandan Women’ (PRIMAL) study aimed to assess the effectiveness of an enhanced HIV counseling intervention for preventing HIV acquisition among HIV-uninfected mothers during pregnancy and throughout the breastfeeding period.

**Methods:**

We conducted an unblinded randomized control trial between 22 February 2013 and 22 April 2016 to assess the effectiveness of an extended repeat HIV testing and enhanced counseling (ERHTEC) intervention aimed at preventing primary HIV infection among HIV-uninfected pregnant and lactating women in Uganda. HIV-uninfected pregnant women aged 15–49 were enrolled 1:1 individually or in couples together with their partner. Enrolled women and couples were randomized 1:1 to an intervention (ERHTEC) or control (extended repeat HIV testing and standard counseling) group and followed up to 24 months postpartum or six weeks past complete cessation of breastfeeding, whichever came first. Both groups were tested for sexually transmitted infections (STIs) and HIV at enrollment, delivery, 3 and 6 months postpartum and every 6 months thereafter until the end of follow-up. The intervention group received enhanced HIV prevention counseling every 3 months throughout follow-up. The control group received standard counseling at the time of HIV retesting. Both intervention and control couples were offered couple HIV testing and counseling at all study visits.

**Main outcome measures:**

Frequency of condom use and incidence of HIV, syphilis, gonorrhea, chlamydia and trichomoniasis over follow-up.

**Results:**

Between February 2013 and April 2014, we enrolled 820 HIV-uninfected pregnant women presenting for antenatal care individually (n = 410) or in couples (n = 410 women and 410 partners) in one urban and one rural public Ugandan hospital. Women’s median age was 24 years (IQR 20–28 years). At baseline, participants did not differ in any socio-demographic, reproductive health, HIV testing history, sexual behavior, medical history or STI status characteristics; 96% (386/402) of couples were tested and counseled for HIV together with their partners at enrolment, 2.1% (7/329) of whom were found to be HIV-infected. Six hundred twenty-five (76%) women completed follow-up as per protocol (S1 Protocol). Women were followed for an average of 1.76 years and cumulated 1,439 women-years of follow-up or 81% of the maximum 1,779 women-years of follow-up assuming no dropouts. Men were followed for an average of 1.72 years.

The frequency of consistent condom use and the proportion of women who used condoms over the last 3 months or at last vaginal sex increased substantially over follow-up in both arms, but there were no statistically significant differences in increases between the intervention and control arms.

During follow-up, on average 42% (range 36%-46%) of couple partners were counseled together. Between 3.8% and 7.6% of women tested positive at any follow-up visit for any STI including syphilis, gonorrhea, C. trachomatis or T. vaginalis. Four women (two in each arm) and no enrolled men became infected with HIV, representing an overall HIV incidence rate of 0.186 per 100 person-years. Three of the women seroconverters had enrolled individually, one as a couple. At or before seroconversion, all four women reported their partners had extramarital relationships and/or had not disclosed their suspected HIV-infected status. There were no statistically significant differences between study arms for STI or HIV incidences.

**Conclusions:**

A sustained enhanced HIV prevention counseling intervention for up to 2 years postpartum among pregnant and breastfeeding women did not have a statistically significant effect on condom use or HIV incidence among these women. However, in both study arms, condom use increased over follow-up while STI and HIV incidence remained very low when compared to similar cohorts in and outside Uganda, suggesting that repeat HIV testing during breastfeeding, whether with enhanced or standard counseling, may have had an unintended HIV preventive effect among pregnant and lactating women in this setting. Further research is needed to verify this hypothesis.

**Trial registration:**

ClinicalTrials.gov NCT01882998

## Introduction

Mother-to-child transmission (MTCT) of HIV in sub-Saharan Africa accounts for 90% of pediatric HIV infections and nearly 10% of all new HIV infections worldwide.[1] While great progress has been made in identifying and treating HIV-infected mothers and preventing the vertical transmission of HIV,[1] the vast majority of the millions of pregnant women who test for HIV every year are HIV-uninfected. Keeping these women uninfected throughout pregnancy and lactation is the first pillar of the World Health Organization (WHO) global strategy for the prevention of mother-to-child transmission (PMTCT)[2] and a key component of the global efforts to eliminate MTCT.[3]

In sub-Saharan Africa, HIV-uninfected pregnant women are at continuous risk of HIV acquisition during pregnancy and breastfeeding with HIV incidence rates ranging between 2 and 14 per 100 women-years. [4–11]. Acquiring HIV during pregnancy or breastfeeding puts mothers at increased risk of adverse health and pregnancy outcomes and exposes their unborn or breastfeeding babies to higher risk of HIV infection because of the high levels of viremia that follow incident HIV infection.[9, 12–17] For these reasons, WHO recommends that pregnant women who test HIV negative at their first antenatal care (ANC) visit be retested in their third trimester in order to identify intercurrent infections and initiate antiretroviral PMTCT prophylaxis as soon as possible.[18] However, this recommendation has not been widely implemented.[19–22] Moreover, the recommendation does not address the risk of incident HIV infection of mothers during the breastfeeding period [9] which, in resource-limited settings, is recommended for as long as needed and desired, and can often last up to 2 years.[18]

African women in general and pregnant women in particular are heavily influenced by their male partners when choosing and implementing HIV risk reduction practices.[23, 24] Couples’ HIV testing and counseling (CHTC) increases uptake of HIV prevention and care services and is an effective strategy for identifying HIV sero-discordant couples and availing them with all options to prevent horizontal and vertical HIV transmission.[25–27] Models of couple mobilization and CHCT have been implemented [28–31], but the effect of these models on sexual risk behavior and HIV transmission has not been systematically evaluated in postpartum breastfeeding women. Evidence about the possible impact repeat HIV testing and counseling (HTC) may have on risky sexual behavior is variable. A randomized controlled trial in serodiscordant couples in Malawi showed that repeated CHTC may promote safer sexual behavior.[32] However, a systematic review on the behavioral impact of finding out one’s own HIV-negative serostatus in sub-Saharan Africa concluded that, with the exception of serodiscordant couples, there is variable evidence that awareness of one's HIV-negative serostatus leads to substantial changes in risk behavior.[33] Data on the potential behavioral effect of repeat testing and counseling in postpartum women are lacking. We hypothesized that: 1) extended repeat HIV testing and enhanced counseling during late pregnancy and throughout breastfeeding can increase and sustain risk reduction behaviors and prevent incident sexually transmitted infections (STI) and HIV infections among HIV-uninfected pregnant and lactating women, and 2) that enhanced couple counseling can further enhance this effect through improved couple communication and emotional and economic support from male partners.

## Materials and methods

We conducted an unblinded parallel randomized control trial in Uganda between 22 February 2013 and 22 April 2016 at Mulago National Referral Hospital in the capital city of Kampala, and St Joseph’s Hospital in Kitgum, a small rural town 430 kms north of Kampala by road.

### Enrolment

We enrolled HIV-uninfected pregnant women of any gestational age individually or with their partners when presenting in couples for their ANC visit. Enrolment started on 22 February 2013 and ended on 18 April 2014 at both study sites. Following protocol, we enrolled an equal number of pregnant women (without their partners) and couples at each site and randomly assigned women or couples 1:1 to an extended repeat HIV testing and enhanced counseling (ERHTEC) intervention or an extended repeat HIV testing and standard counseling control group. Inclusion criteria for women were: age 18–49 years; confirmed pregnancy (at any gestational age); documented HIV-negative sero-status at the time of screening; and intention to breastfeed. Partners’ inclusion criteria were: age ≥18 years; being recognized by the eligible pregnant woman as her current husband/partner for at least 3 months. Additional inclusion criteria for all included: a) residing within 30 km of the study clinic and not planning to move; b) agreeing to come the study clinic for scheduled appointments, to undergo study procedures, and to be called or visited at home by trained home visitors as needed to ensure retention; c) absence of medical condition likely to interfere with participants’ ability to adhere to study procedures; and e) providing written informed consent.

Pregnant women were tested routinely for HIV at their first ANC visit by rapid HIV testing as per Uganda National PMTCT Program Guidelines.[34]. Women presenting in couples were counseled together with their partners or separately as per their choice. Clinic midwives referred women testing HIV-negative to study counselors and research assistants. Study staff reviewed study eligibility criteria, assessed participants’ interest in the study and read the informed consent forms to potential participants. Consenting participants were retested for HIV by rapid testing and women and couples whose female partner re-tested HIV-negative were referred to the study clinic. From that point on, all subsequent procedures were carried out separately for women and men except when couples opted to undergo CHTC. At enrolment, all participants (women and partners) were examined physically, tested for syphilis, gonorrhea, chlamydia, and *T*. *vaginalis*, and administered baseline behavioral questionnaires about socio-demographic and behavioral characteristics, including partner communication and support, sexual behavior, and family planning. All questionnaires were administered by trained research assistants in the local languages of participants (Luganda or Luo) or in English if preferred. Study counselors did not administer behavioral questionnaires to avoid conflicting their relationship with participants. Data were collected on Samsung Galaxy Note 10.1 tablets where Luganda, Luo and English versions of the questionnaires had been entered and logically checked using version 1.0.2 of the Open Data Kit (ODK) open-source software.

### Follow-up and procedures

Follow-up visits were scheduled for all participants (women and their partners) around the estimated time of labor and delivery, as well as at 3, 6, 12, 18 and 24 months postpartum (in alignment with Uganda’s routine post-natal visit schedule up to 18 months), or until 6 weeks after complete cessation of breastfeeding, whichever occurred first. At each of these visits, all participants (women and men) were examined physically for STI symptoms, tested and counseled for HIV, and were administered a follow-up behavioral questionnaire (separately for men and women) focusing on changes in demographics, sexual behavior, partner communication and support, family planning, and relational scales. All women and symptomatic men were bled for syphilis and HIV rapid testing and vaginal or urethral swabs were collected for gonorrhea, chlamydia and *T*. *vaginalis* testing. The intervention group were also seen at 9, 15 and 21 months postpartum for enhanced prevention counseling. At the end of follow-up, all study participants received enhanced counseling irrespective of their arm assignment received.

Study participants were followed in a dedicated clinic at each study site for all scheduled and unscheduled visits. The clinic was open 5 days a week with a 24/7 dedicated telephone line available for participants to call for any reason. Study home visitors called participants daily to remind them of their appointment date and or to find out why they did not show up for a given visit. Participants who could not be reached by phone were visited at home. All participants were reimbursed the equivalent of US $3.00 for travel expenses to attend scheduled follow-up visits. Follow-up ended on April 22, 2016 in both sites.

At each scheduled postnatal visit, women were asked if they were still breastfeeding. Women reporting they were no longer breastfeeding were asked when they had stopped breastfeeding and if they had stopped completely or partially. Only women reporting having stopped completely for the prior six weeks or more with no intention to resume at a later date were considered having reached the end of breastfeeding on the date this information was obtained from the mother, or the date of the last study visit at which the woman was seen if she had never declared an end to breastfeeding.

#### Intervention

Both individual and couple ERHTEC groups received enhanced HIV prevention counseling by trained study counselors at enrolment, around labor and delivery, and every 3 months thereafter throughout follow-up. We developed a specific guide for the enhanced counseling intervention focusing on primary HIV prevention in relation to the specific contexts and risks of incident infection for pregnant and lactating women, all of which were not covered in the national or international guidelines.[35, 36] In addition to integrating safe motherhood, safe infant feeding practice and family planning guidance found in the national guidelines, the guide clarified the concepts of acute and incident infection, explained the HIV testing “window” period, and described risk behaviors and sources of potential exposure to HIV between repeat HIV tests. Specific vulnerabilities and risks were discussed at different stages of pregnancy, delivery, and the post-partum period, with a particular focus on cultural practices and beliefs associated with sexual activity during pregnancy and breastfeeding and resumption of sexual activity after delivery. The guide also added a section specific to pregnant and post-partum couples in complement to the Couple HTC Guidelines in use in Uganda at the time,[37] This section emphasized couple communication, sexual behavior and negotiation, HIV status disclosure during pregnancy and lactation, the implications of serodiscordance including becoming newly serodiscordant, and all means to minimize transmission risks. Lastly, the guide also addressed common mental health conditions pregnant and post-partum women and their partners may experience, including identification and referral for treatment of post-partum depression, all of which were not part of the national guidelines.

The control group received repeat HIV testing and standard counseling from clinic nurses as available at scheduled follow-up visits. Standard counseling content was expected to follow Uganda National Implementation Guidelines for HIV Counselling and Testing.[37] Briefly, this included at a minimum: assessing client’s readiness to receive the HIV test result; giving the test result clearly and unambiguously, assessing the client’s understanding of the test result and its implications; making plans for risk reduction, partner notification and testing, involving significant others in disclosure; making arrangement for follow-up support; and making plans for referral and linkage to prevention, treatment, care and support services as needed. [37]

Primary outcome measures included the frequency of condom use and incidence of HIV, syphilis, gonorrhea, chlamydia and trichomoniasis over follow-up. Condom use was documented through self-report.

#### Diagnosis and management of incident HIV and STI infections

HIV testing followed the national and WHO algorithms in force at the time of the study [37, 38], using three sequential but distinct third-generation antibody rapid tests (Abbott Determine Rapid HIV-1 Test, Abbott Laboratories, Lake Bluff, Illinois, USA; Stat-Pak Rapid HIV-1 Test, Chembio Diagnostic Systems, Medford, NY, USA; and Unigold HIV Rapid Test, Trinity Biotech, Bray, Ireland).

In case of incident HIV antibody seroconversion, the reactive sample as well as all stored study participants serum samples from every prior visit since enrolment were retested for HIV DNA by polymerase chain reaction (PCR) assay at the Uganda Virus Research Institute. Samples reactive on HIV DNA PCR were considered confirmed for HIV infection. Participants were considered infected at the time of, or prior to, their earliest PCR-reactive sample.

Rapid syphilis, gonorrhea and chlamydia diagnostic testing was performed systematically for all women and for symptomatic men at each scheduled visit using lateral flow chromatographic sandwich antibody or antigen immunoassays (One Step Strip Syphilis rapid antibody test, One Step Strip Gonorrhea rapid antigen test, and One Step Cassette Chlamydia rapid antigen test, PreChek Bio, Anaheim, CA, USA). Serum was used for the One Step Strip Syphilis test while vaginal and urethral swabs were used for the One Step Strip Gonorrhea and Cassette Chlamydia rapid antigen tests for women and men, respectively. Freshly collected swab samples were extracted following the manufacturer’s instructions, stored at 4–8°C and tested within 24 hours at room temperature. Testing followed standard procedures consisting in immersing strips into serum or extracted swab samples for 5 seconds or adding 150ul of serum or extracted swab samples to the sample well of testing cassettes. Results were read after 10–15 minutes; the appearance of two distinct colored bands representing the control and tested sample bands indicated a positive test result. Testing for the presence of *T*. *vaginalis* relied on the wet mount microscopy technique using fresh vaginal and urethra swab samples.[39]

All participants diagnosed with an incident STI or HIV infection during follow-up received standard of care treatment in accordance with Uganda’s National STI Treatment Guidelines and Uganda Integrated Guidelines on ART, PMTCT & Infant and Young Child Feeding.[35, 40] Partners of participants found to be infected with an STI were notified and offered treatment as per national guidelines.[40]

In case of incident HIV infection identified during pregnancy, labor and delivery or while breastfeeding, women participants received standard care as per the WHO/MOH PMTCT guidelines in use since 2012, which consisted in initiating lifelong ART (Option B+) upon HIV infection diagnosis irrespective of CD4 count or WHO clinical stage.[41] In addition, all HIV-exposed infants (babies born to HIV-infected mothers) received daily nevirapine through age 4–6 weeks regardless of the infant feeding method. HIV-exposed babies ≥6 weeks of age were tested by HIV DNA PCR and if confirmed to be infected, were initiated on triple ART. Uninfected infants were retested at 18 months of age or ≥6 weeks after complete cessation of breastfeeding, whichever occurred first, and started on ART if infected.

#### Adverse events and study withdrawal

Adverse events were investigated, monitored and reported to the local and international Institutional Review Boards (IRBs) following the International Conference on Harmonization Guideline Criteria.[42] The main risks to participants were involuntary of forced HIV status disclosure and intimate partner violence (IPV). In either case, women or couples were offered counseling by study counselors and referred to a hospital for physical examination and to local organizations experienced in dealing with IPV-associated trauma. Women participants could be withdrawn from the study for one of the following reasons: withdrawing consent, moving permanently outside the study area, missing two consecutive scheduled follow-up study visits, or death of their infant. Male partners’ exclusion from the study was contingent on women’s exclusion but not vice-versa, i.e. couples were withdrawn from the study only if the woman was withdrawn; however, women were not withdrawn if their male partner defaulted from any number of follow-up study visits. This was done to promote adherence among non-conflicted couples and to allow time for support counseling for conflicted couples. Participants who exited the study 3 weeks or more after their last attended scheduled visit were invited for an end-of-study visit where they received all intervention procedures irrespective of their original arm assignment.

### Sample size, randomization, and allocation

We calculated target sample sizes to provide 80% power in 2-sided tests with a type-1 error rate of 5% to detect a 10% reduction in the frequency of unprotected vaginal intercourse in the treated group of women independent of their enrolment site or category (individual or couples) relative to controls, accounting for within-subject correlation and loss to follow-up. Our calculations assumed a Poisson distribution with 2-fold over-dispersion, within-subject correlation of the repeated measures of 0.3, a baseline frequency of 5 sexual encounters per months, a post-baseline reduction in frequency of unprotected vaginal intercourse of 30% among controls, and up to 50% loss to follow-up occurring gradually over 2 years. These calculations were done in R (R Foundation for Statistical Computing, Vienna, Austria). Numbered randomization lists were computer-generated off site by the study statistician and allocated sequentially by the study coordinators. Intervention assignments were not blinded to the study staff or the participants.

### Statistical analysis

We treated all available outcomes measured at serial visits as repeated binary or ordinal measures, and used the visit type at which each outcome was obtained as part of the analysis up to the last visit before or at termination, withdrawal, or completion of follow-up. Participants did not contribute outcomes beyond these time points. Study participants who exited the study for any reason contributed outcomes for all analyses up until their last completed follow-up or end-of-study visit.

We used generalized estimating equations (GEE) models with robust standards to assess treatment effects on repeated binary or ordinal outcomes ascertained at baseline and up to 6 follow-up visits. Linear, logistic, binomial, proportional odds, and negative binomial GEE models were used as appropriate to fit the distribution of each outcome. Two models were estimated for each outcome. The first included indicators for visit, treatment assignment, and the interaction of treatment with follow-up vs baseline visit; the interaction captured the treatment effect on average change since baseline. The second model was restricted to follow-up visits and included indicators for visit and treatment assignment; this model captured the average between-group difference. Person-time for incident HIV or STI infections was calculated by cumulating the number of person-years of follow-up contributed by each newly infected participant. Participants could contribute repeated STI events during that time except for syphilis for which only the first positive test was counted as a new case since 85% of positive Treponemal antibody tests can remain reactive for a patient’s lifetime regardless of treatment.[43]

### Ethical approval

The study was approved by the IRB of Makerere University School of Medicine on 17 September 2012, Kampala, Uganda, the Uganda National Council of Science and Technology on 16 October 2012, and the Committee on Human Research of the University of California San Francisco, USA on 18 December 2012.

### Trial registration

This study was registered in *ClinicalTrials*.*gov* (www.ct.gov) under registration number NCT01882998. Submission to ct.gov was initiated in June 2012 but not completed in June 2013 as the system kept generating an error that required the system administrator’s intervention. The authors confirm that all ongoing and related trials for this intervention are registered.

## Results

### Trial profile

**Fig 1:**
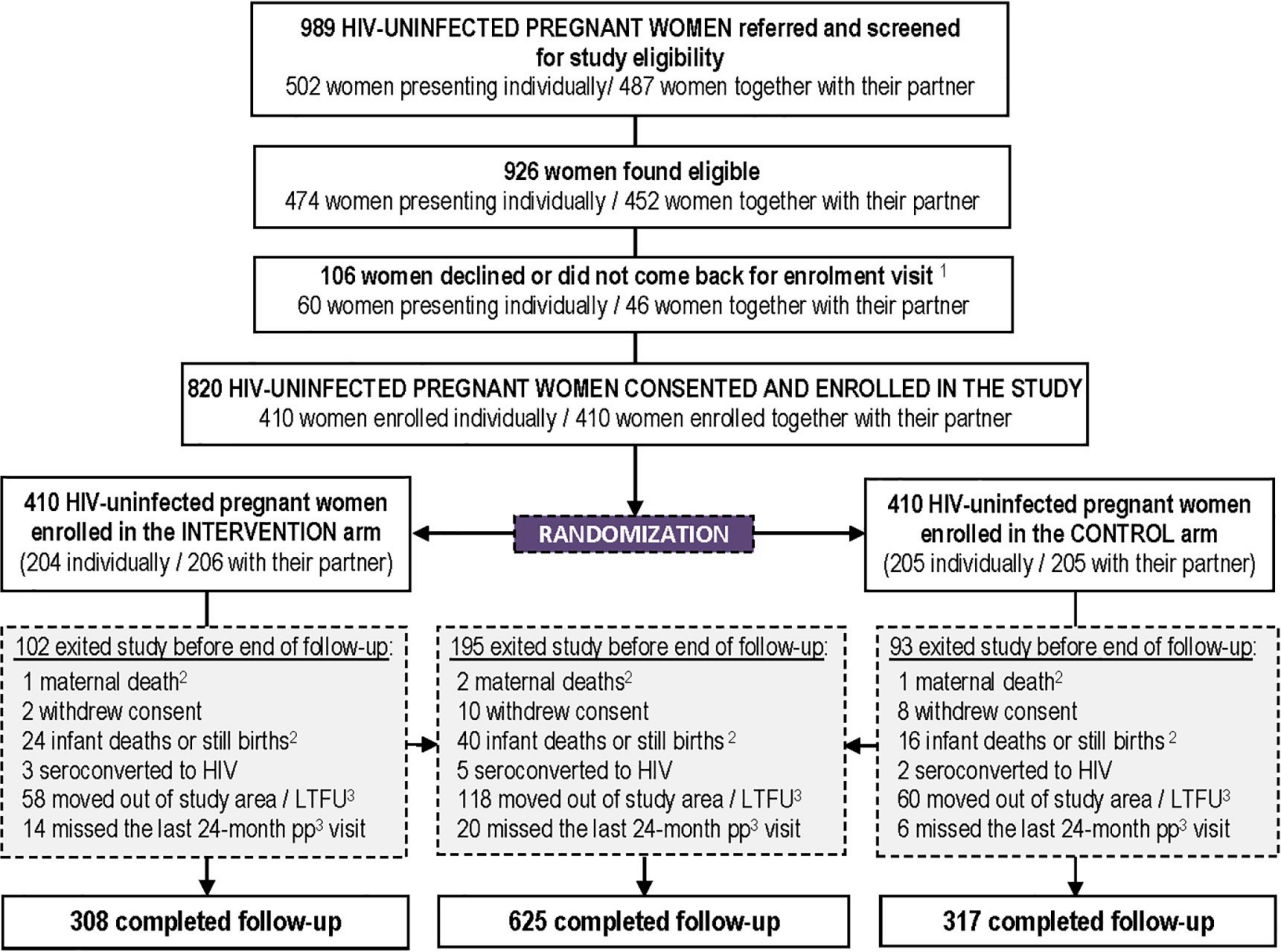
Trial profile. ^1^ None of these women presented with any particular risk profile according to their screening data; ^2^ Unrelated to the intervention ^3^ LTFU: lost to follow-up.

Between 22 February 2013 and 18 April 2014, 989 HIV-negative pregnant women presenting individually (n = 502) or with their partners (n = 487) at the ANC clinics of both study sites were referred to the study clinics for eligibility assessment. Of these, 94% (926/989) women were found eligible, and 89% (820/926) were enrolled in the study; 410 with their partner and 410 without their partner. Randomization assigned 204 women enrolled individually and 206 couples to the intervention arm, and 205 women enrolled individually and 205 couples to the control arm (Fig 1). These 820 randomized women were followed for an average of 1.76 years, totaling 1,439 or 81% of the maximum 1,779 women-years of follow-up. Mean pre-partum follow-up was 0.32 years, totaling 265 women-years of follow-up while mean post-partum follow-up was 1.43 years totaling 1,173 women-years of follow-up or 81% of the maximum number women-years assuming no drop outs. Men were followed for an average of 1.73 years, totaling 709 person-years of follow-up. Women breastfed their babies for a median 645 days or 21.2 months (IQR 17.6–22.1 months) and 625/820 (76%) of enrolled women completed follow-up per protocol; of these, 583 (71%) women (248 in Kampala, 335 in Kitgum) actually attended the 24-month postpartum visit.

### Baseline characteristics

#### Socio-demographic and behavioral characteristics

**Fig 2:**
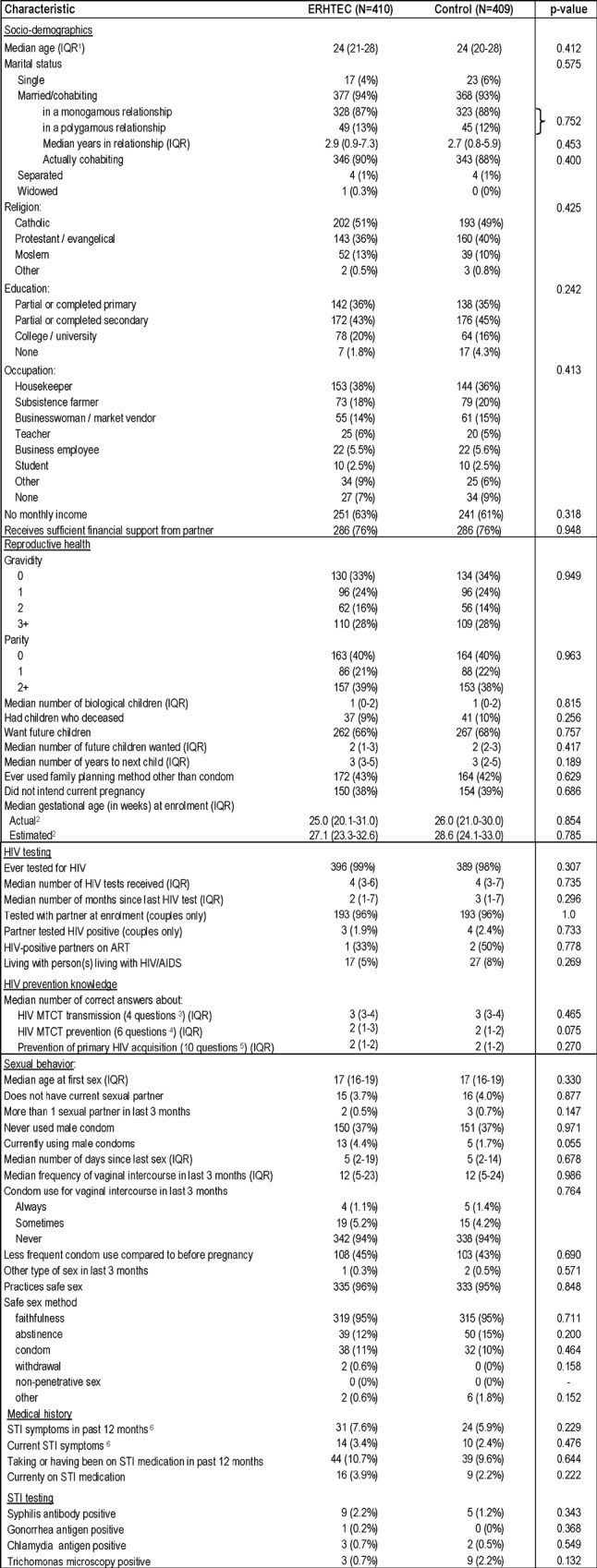
Baseline characteristics of intervention and control women at enrolment. 1. IQR: Interquartile range 2. Actual values are based on 631/818 (77%) of women who had a gestational age captured at enrolment–midwives had no access to ultrasound imaging, had problems with last normal menstrual period recall as well as fundus height measurement. Estimated gestational age was based on time between enrolment and delivery dates and an estimated average pregnancy duration of 40 weeks. 3. Questions on mother-to-child HIV transmission (MTCT) explored knowledge about transmission during pregnancy, delivery, breastfeeding and post-breastfeeding 4. Questions about prevention of MTCT explored knowledge about HV antiretroviral therapy prophylaxis, delivery in health facilities, baby nevirapine prophylaxis, exclusive breastfeeding, duration of breastfeeding, and formula feeding, 5. Questions about prevention of primary maternal HIV acquisition explored knowledge about abstinence, condom use, risk of transfusions and injections, partner testing, breastfeeding avoidance and breastfeeding duration, health facility delivery, faithfulness, and testing for HIV. 6. Symptoms probed or examined included: Abnormal vaginal discharge, bleeding, itching, swelling, vesicles, pustules, odor, genital or anal ulcers/sores/warts, frequent or painful urination, pain or bleeding during intercourse, and lower abdominal pain.

There were no statistically significant differences between intervention and control participants for any of their baseline socio-demographics, reproductive health characteristics, HIV testing history, HIV prevention knowledge, sexual behavior characteristics, medical history or STI status (Fig 2). Enrolled women’s median age was 24 years (interquartile range [IQR] 20–28 years); 94% of them were married or cohabiting with their partner, 13% of them were in a polygamous union. Fifty-seven percent of women were in their first pregnancy, and 9% (78/811) had lost one or more children prior to their current pregnancy. Sixty percent had ever used a male condom, and 58% (457/793) had never used a family planning method other than condoms. Almost all women (99%) had been previously tested for HIV; among enrolled couples, 96% (386/402) were tested and counselled for HIV together with their partners, and 2.1% (7/329) of partners were found to be HIV-infected, for which 3 of whom were on ART.

The sexual behavior profiles of women were also very comparable with no statistically significant differences across treatment arms. Only 9 (1.2%) and 34 (4.7%) of 723 women reported using condoms consistently or intermittently respectively for vaginal intercourse in the last 3 months and 96% of women stated they practiced safer sex. When prompted, 95% of these women defined safer sex as faithfulness, 13% as abstinence, 10% as using condoms, and 0.3% as practicing withdrawal (Table 1). Lastly, 3.8% (31/819) of women tested positive for an STI at enrolment (14 syphilis, 12 *T*. *vaginalis*, 5 chlamydia and 1 gonorrhea, including one dual infection).

**Table 1:** Serodiscordant couples (F-M+) at baseline and during follow-up.

Serodiscordance (F-M+)	KITGUM (rural)			KAMPALA (urban)			ALL
	EHRTEC	Controls	Total	EHRTEC	Controls	Total	Totals
**Among male partners of women enrolled individually (as reported by the women)**							
Baseline	2	2	**4**	1	1	**2**	**6**
Follow-up	1	4	**5**	0	0	**0**	**5**
**Total**	**3**	**6**	**9**	**1**	**1**	**2**	**11**
**Among male partners enrolled with their wife (through HIV testing)**							
Baseline	0	2	**2**	4	3	**7**	**9**
Follow-up	0	0	**0**	0	0	**0**	**0**
**Total**	**0**	**2**	**2**	**4**	**3**	**7**	**9**

### Efficacy of ERHTEC intervention on sexual activity, condom use, HIV and STI prevention

#### Sexual activity

**Fig 3:**
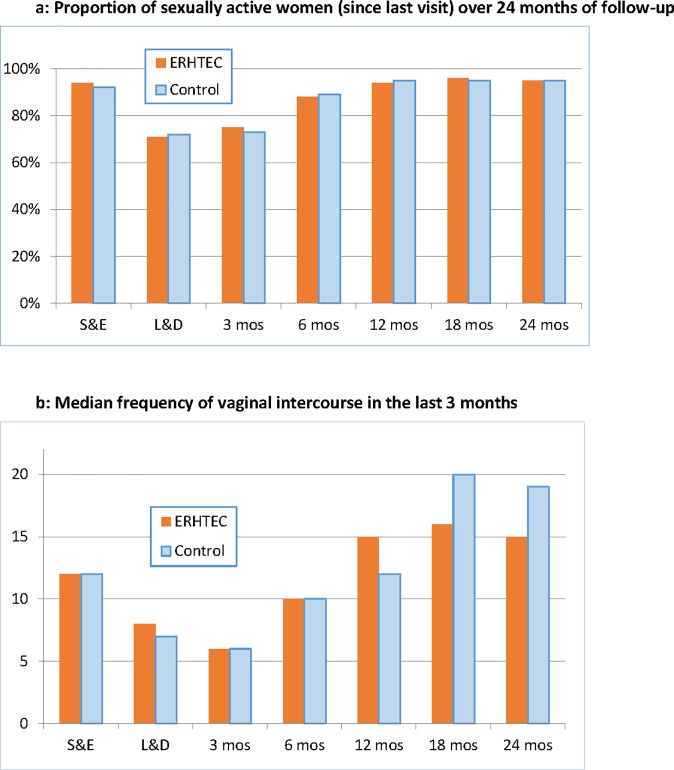
**a. Proportion of sexually active women since last visit, by visit** Difference in average change by treatment assignment: p = 0.120 Difference in average follow-up level by treatment assignment: p = 0.952 **b. Median frequency of vaginal intercourse in the last 3 months, by visit** Difference in average change by treatment assignment: p = 0.615 Difference in average follow-up level by treatment assignment: p = 0.592.

There were no statistically significant difference in sexual activity between the intervention and control arms over follow-up (Fig 3A and 3B), For both groups, both the proportion of women reporting sexual activity and the median frequency of vaginal intercourse in the last 3 months decreased from enrolment to the time of labor and delivery through 3 months postpartum and resumed steadily thereafter.

#### Condom use

**Fig 4:**
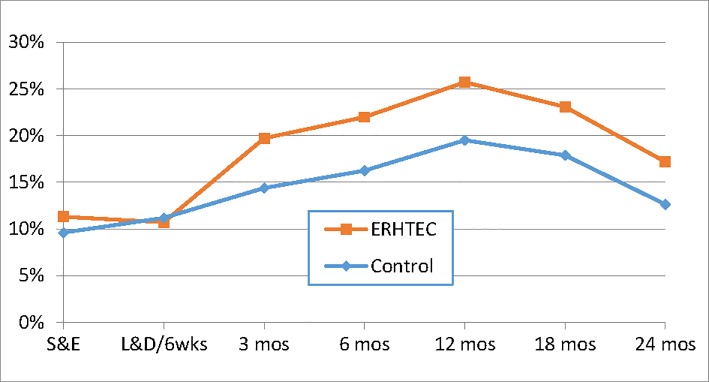
Proportion of women who practiced safer sex through condom use in the last 3 months, by visit. Difference in average change by treatment assignment: p = 0.665 Difference in average follow-up level by treatment assignment: p = 0.026.

At each follow-up visit, women were asked if and how they were practicing safer sex. At every visit, between 91% and 98% of women in both groups responded they were practicing safer sex (S1 Fig). Of these, 93% to 97% of women in both arms said they were practicing safer sex through faithfulness. There were no statistically significant differences between the intervention and control groups. Starting at 3 months postpartum to the end of follow-up, a consistently higher proportion of women in the intervention group reported condoms as their safer sex method compared to women in the control group, reaching 25.7% (71/276) vs 19.5% (55/282) at 12 months postpartum (Fig 4); this difference was statistically significant in average follow-up level (p = 0.026) but not in average change (p = 0.665) by treatment assignment.

**Fig 5:**
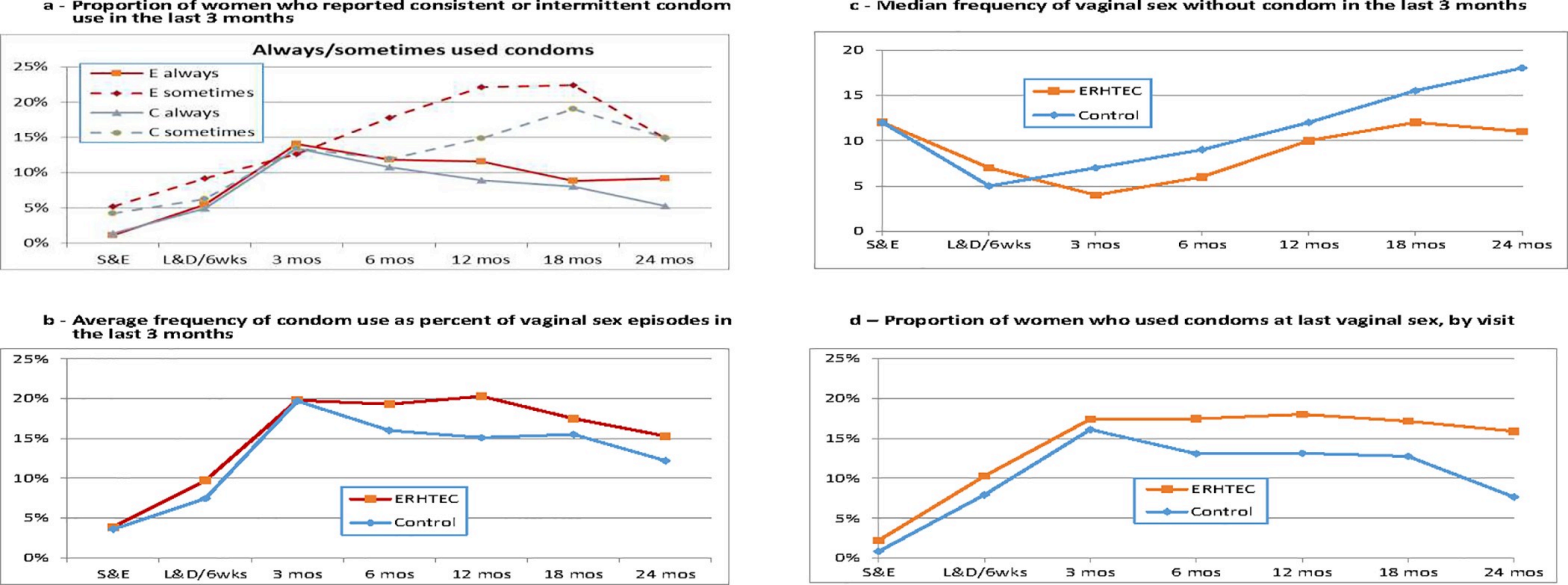
**a—Proportion of women who reported consistent or intermittent condom use in the last 3 months, by visit** Difference in average change by treatment assignment: p = 0.665 Difference in average follow-up level by treatment assignment: p = 0.056 **b—Average frequency of condom use as percent of vaginal sex episodes in the last 3 months** Difference in average change by treatment assignment: p = 0.209 Difference in average follow-up level by treatment assignment: p = 0.137 **c—Median frequency of vaginal sex without condom in the last 3 months** Difference in average change by treatment assignment: p = 0.534 Difference in average follow-up level by treatment assignment: p = 0.148 **d—Proportion of women who used condoms at last vaginal sex** Difference in average change by treatment assignment: p = 0.353 Difference in average follow-up level by treatment assignment: p = 0.029.

Women who were sexually active in the last 3 months and reported to use condoms were also asked whether they used them consistently, intermittently or never (Fig 5A). While fewer than 6% at enrollment, respondents reporting consistent condom users increased steadily through 3 months postpartum, while intermittent condom users increased steadily through 18 months postpartum among women in both arms (Fig 5A). The proportion of women reporting consistent condom use increased from 1.1% (4/365) and 1.4% (5/358) at enrolment to 14.0% (31/221) and 13.5% (28/208) at 3 months postpartum in the intervention and control groups respectively. Thereafter, the proportion of consistent condom users decreased gradually through the end of follow-up in both groups to 9.2% (24/262) and 5.2% (14/267) respectively at 24 months. Similarly, the proportion of women reporting intermittent condom use increased from 5.2% (19/365) and 4.2% (15/358) at enrolment to 22.4% (59/237) and 19.1% (45/236) at 18 months postpartum respectively, and this increase was more sustained in the intervention than in the control group (Fig 5A). However, the statistical difference in average change and follow-up level by treatment assignment between the study arms failed to reach significance (p = 0.665 and 0.055 respectively—Fig 5A).

We also examined the frequency of condom use for vaginal sex in the last 3 months (Fig 5B and 5C), expressed as the proportion of vaginal sex episodes with a condom at a given follow-up visit. The frequency of condom use for vaginal sex increased in both study arms from enrolment until labor and delivery (Fig 5B). Frequency of use went from less than 4% at enrolment to nearly 20% at 3 months postpartum. Thereafter, it remained stable through 12 months postpartum in the intervention group then decreased to 15% by the end of follow-up, while it decreased constantly in the control group to 12% by the end of follow-up (Fig 5B). These differences did not reach statistical significance (p = 0.209 and 0.137 for differences in average change and follow-up level by treatment assignment respectively, Fig 5B).

Finally, we looked at condom use at last vaginal sex (Fig 5D). Here again, the proportion of women in both arms reporting to have used a condom at last sex steadily increased from enrolment (2.2% [8/361] and 0.8% [3/353] for intervention and control women respectively) to 3 months postpartum (17.4% [39/224] and 16.1% [34/211] respectively). From that point on, the proportion of intervention women reporting condom use at last sex remained stable while it decreased among control women to 7.7% (21/274) through 24 months postpartum. This difference was statistically significant by average follow-up level (p = 0.029) but not by average changes by treatment assignment (p = 0.353) (Fig 5D).

Sub-analyses of the variables above stratified by enrolment status (individual vs couple) or site assignment (urban vs rural), or restricted to women who reported not using condoms at baseline, did not reveal statistically significant differences either between the two arms of the study (S2–S7 Figs, S2 Table)

#### STI incidence

**Fig 6:**
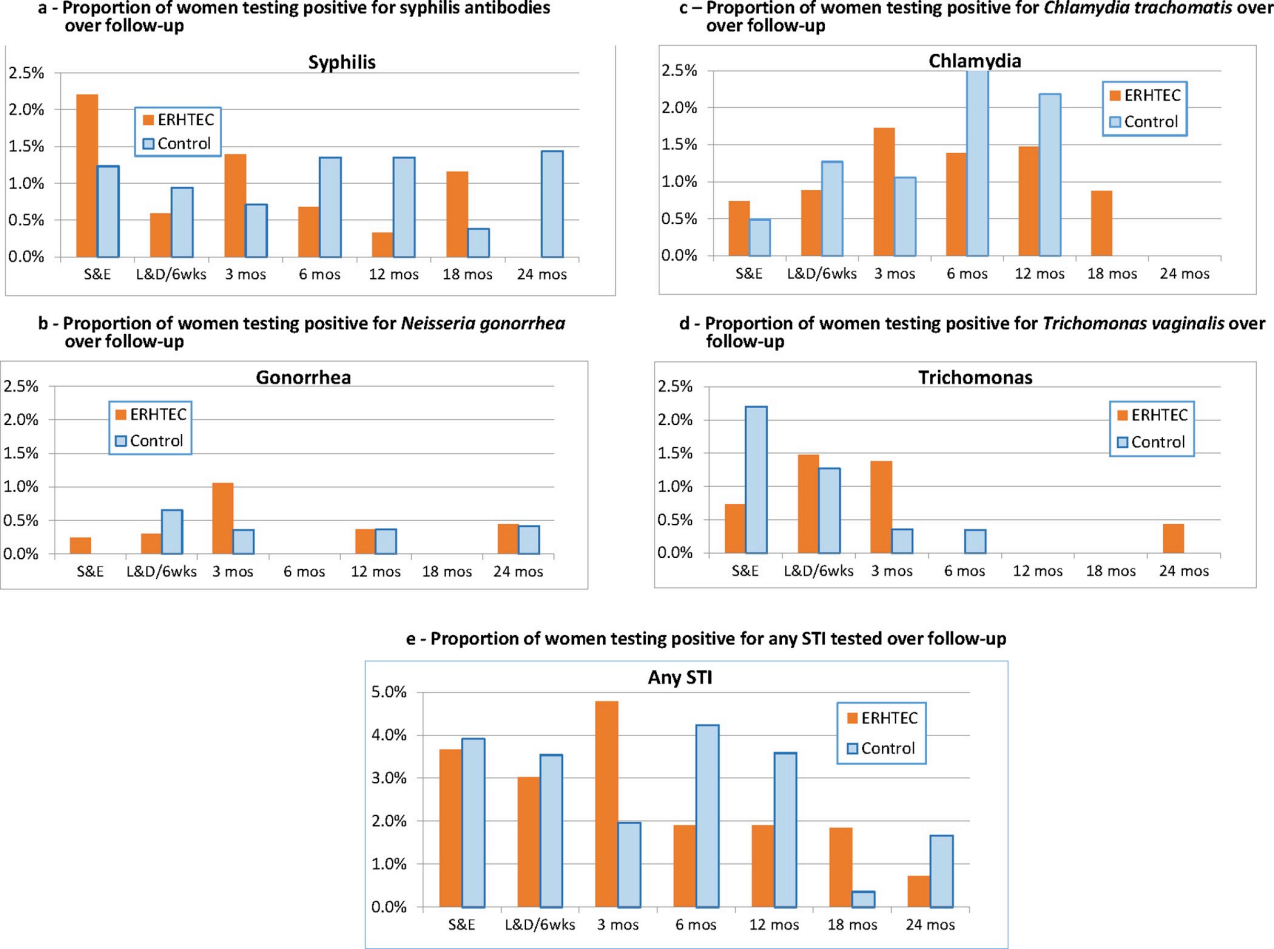
**a. Proportions of women testing positive for syphilis antibodies over follow-up** Difference in average change by treatment assignment: p = 0.136 Difference in average follow-up level by treatment assignment: p = 0.266 **b. Proportions of women testing positive for *Neisseria gonorrhea* over follow-up** Difference in average change by treatment assignment: p = 0.632 Difference in average follow-up level by treatment assignment: p = 0.785 **c. Proportions of women testing positive for *Chlamydia trachomatis* over follow-up** Difference in average change by treatment assignment: p = 0.543 Difference in average follow-up level by treatment assignment: p = 0.595 **d. Proportions of women testing positive for Trichomonas vaginalis over follow-up** Difference in average change by treatment assignment: p = 0.051 Difference in average follow-up level by treatment assignment: p = 0.367 **e. Proportions of women testing positive for any of the STIs tested over follow-up** Difference in average change by treatment assignment: p = 0.681 Difference in average follow-up level by treatment assignment: p = 0.753.

At enrollment, 70 women (8.5%) reported having been in a sexual relationship with a person with a known STI in the past 12 months while 33 (4%) reported being in a current relationship with a person with a known STI. At baseline, 14 women (1.7%) tested positive for syphilis, one for gonorrhea, five (0.6%) for chlamydia and 12 (1.5%) for *T*. *vaginalis*. Prior to delivery, two women (0.3%) newly tested positive for syphilis, three (0.5%) for gonorrhea, seven (1.1%) for chlamydia, and nine (1.4%) for *T*. *vaginalis*. During postpartum follow-up, 34 additional women (1.2%) newly tested positive for syphilis, eight (0.3%) for gonorrhea, 32 (1.2%) for chlamydia, and seven (0.3%) for *T*. *vaginalis* (Fig 6A–6E). Overall incidence rates were 3.5 per 100 women-years (WY) for syphilis, 0.8 per 100 WY for gonorrhea, 3.1 per 100 WY for chlamydia, and 1.9 per 100 WY for *T*. *vaginalis*. There were no statistically significant differences between the intervention and control arms in STI prevalence at baseline, or incidence over follow-up (all p-values > 0.05). Sub-analyses of STI incidences by enrolment status (individual vs couple) or site assignment (urban vs rural) did not reveal statistically significant differences between the two arms (S3 Table).

#### HIV incidence

At enrollment, six women enrolled individually reported to be in an HIV-serodiscordant relationship while nine enrolled with a partner who tested HIV positive (Table 1), No enrolled men was found to seroconvert during 709 person-years of follow-up, and no HIV transmission was found to occur among the nine enrolled serodiscordant couples over 15.6 person-years of follow-up (Table 1).

Six women seroconverted during follow-up (Table 2). The HIV status of the male partners of three out of the four women enrolled individually was unknown at enrolment while the two male partners enrolled with their wife were both HIV-negative at enrolment. However, the partner of woman number 5 in Table 2 had last tested for HIV eight months before his wife seroconverted after which he stopped attending follow-up visits.

**Table 2:** Women who seroconverted for HIV during follow-up.

Enrolment group, arm and site	Sero-conversion date	Sero-conversion visit	HIV DNA PCR test result	Final HIV status	Partner’s HIV status at enrolment	Partner HIV status	Partner’s last HIV test date	Infant HIV PCR test result
*1. Indiv-ERHTEC-KA*	*25-Oct-2013*	*L&D*	*POS at S&E*	*Infected at enrolment*	*Unknown*	*n/a*	*n/a*	*n/a*
*2. Couple-CTRL-KI*	*11-Nov-2014*	*12m*	*NEG*	*Uninfected*	*NEG*	*NEG*	*20-Jul-15*	*NEG*
3. Indiv-ERHTEC-KI ^**a**^	7-Apr-2015	12m	POS	Seroconverted	NEG	POS	15-Mar-14	NEG
4. Indiv-CTRL-K I ^**b**^	18-Jun-2015	21m	POS	Seroconverted	Unknown	POS	15-Jun-15	NEG
5. Couple-ERHTEC-KI ^**c**^	14-Jul-2015	12m	POS	Seroconverted	NEG	NEG	20-Nov-14	NEG
6. Indiv-CTRL-KI	22-Oct-2015	18m	POS	Seroconverted	Unknown	POS	15-Oct-15	NEG

Abbreviations: EHRTEC: Intervention; CTRL: Control; KA: Kampala; KI: Kitgum; L&D: Labor and delivery; NEG: Negative; PCR: Polymerase chain reaction; POS: Positive; S&E: Screening and enrolment

a) While woman number 3 reported her non-enrolled husband’s HIV status as negative at enrolment, during her post-seroconversion counseling session, she reported that he had been diagnosed as HIV-positive about a year ago but had not disclosed to her and had been poorly adherent to ART.

b) After multiple counseling sessions following her seroconversion, woman number 4 enrolled individually who lived in a polygamous relationship came to re-test for HIV together with her husband and two co-wives. The study participant, her husband and one co-wife tested HIV-positive and were given treatment; the other co-wife tested HIV-negative.

c) Woman number 5 and her partner both tested HIV-negative at enrolment but the partner tested positive for syphilis and was given treatment but it is unclear he took it. However, the couple had separated by the time of their 6-month postpartum visit; she then reported she became increasingly distrustful of him because he had many sexual partners. The partner never came back after this visit.

HIV DNA PCR testing of the first seroconverter’s blood samples collected since enrolment found all of them to be HIV PCR positive, showing she was already infected at enrolment while in the window period. She was therefore not counted as a seroconverter and referred back to the ANC clinic where she was enrolled in care through the national PMTCT program. The blood sample of the second woman who seroconverted by rapid HIV testing, as well as two separate subsequent blood samples collected two months past her seroconversion date, could not be confirmed by HIV DNA PCR despite multiple retests or Western blots performed in two separate laboratories (Table 2). Her final status was therefore considered HIV-uninfected. She thus continued in the study and remained clinically well until completing follow-up per protocol 9 months later in July 2015 at her 18-month post-partum visit. The remaining four seroconverters—all from the rural (Kitgum) study site—were confirmed to have acquired HIV during follow-up as their seroconverting samples tested positive by HIV DNA PCR while all their previous follow-up samples remained HIV DNA negative. These seroconversions were detected at 12-, 18- and 21-month postpartum visits. Two of the four confirmed seroconverters belonged to the intervention arm and two to the control group, while three were enrolled individually and one as couple (# 5, Table 2). Three of these four women were in an unknown partner serostatus relationship at enrolment while the one enrolled in a couple (# 5) had an HIV-negative partner. At the time of the women’s seroconversion, three of the non-enrolled partners (# 3, 4 and 6) were found to have seroconverted while the enrolled partner (# 5) had stopped attending follow-up visits eight months before his wife seroconverted and was last tested HIV-negative at that time.

Together, these four confirmed seroconversions represent an HIV incidence rate of 0.278 (95% CI: 0.08–0.71) per 100 women-years of follow-up among the women cohort and 0.186 (95% CI: 0.00–0.52) per 100 person-years of follow-up among the entire cohort (women and men).

#### Adverse events

Fifty-seven adverse events occurred during the study. Two women, one in the intervention and one in the control arm, died shortly after delivery; both deaths were consequent to postpartum hemorrhage. Forty-six women lost their babies through: 15 still births; 11 neonatal deaths due to severe birth asphyxia, fetal distress, sepsis, or other causes; 13 spontaneous or induced abortions and intrauterine fetal deaths; and 7 infant deaths due to complicated malaria, respiratory or gastro-intestinal infections or other unknown causes. Two male partners died during follow-up; one in a motorcycle accident, the other after falling into an alcohol-induced coma. Seven other adverse events included: thrombosed hemorrhoids, vaginal cystocele, a broken metatarsal, a case of partner violence, a molar (repeat) pregnancy, a postpartum psychosis that was treated successfully, and the theft of a study laptop and a tablet that resulted in recalling 5 participants for repeat data collection. The woman who suffered domestic violence was severely beaten by her husband after she told him to stop drinking and coming back home late at night. The PRIMAL Counselor referred her to a hospital clinician for physical examination and to the African Network for the Prevention and Protection against Child Abuse, a local organization that helped the mother file a police report and return with her children to her parents’ home. She continued to participate in the study without further incidents. None of the events or deaths could be related to the study intervention or services. All events were reported to the study Uganda IRB within 7 days of their occurrence, and to the US IRB and the study sponsor (NIH/NICHD) annually.

## Discussion

This randomized controlled study did not find a clear statistically significant effect of an extended repeat HIV testing and enhanced counselling intervention in increasing condom use or reducing HIV or STI incidence among HIV-uninfected pregnant and lactating Ugandan women participants. However, overall condom use did increase significantly over follow-up in both arms of the study, and the overall HIV and STI incidence rate for this cohort was very low. Several factors may explain this finding. Enhanced counseling may have not been sufficient to induce a significant behavior change among intervention women or their partners. Personal, social and structural factors as well as limited partner involvement may have influenced sexual practices among both control and intervention women that enhanced counseling was not able or sufficient to impact.[44–46] For example, a great majority of women in both study arms considered faithfulness as their primary safer sex practice, a perception that did not change throughout follow-up. This may have constituted an important obstacle for intervention women to heed their counselors’ advice on consistent condom use.[47, 48]

Additionally, whether enrolled as a couple or individually, most women may have not had the ultimate say on using condoms as shown by the absence of significant differences in condom use between women enrolled individually and in couples. In addition to the dominant role traditional Ugandan culture confers to men for sexual matters, this may have been exacerbated by the fact that, despite the inclusion of couples in the trial, less than half of the women enrolled as couples were accompanied by their male partners at any follow-up visit (S1 Table) and thus the majority of these women may not have benefitted from couple counseling, contrary to what was intended.

It is also possible that, irrespective of the enhanced counseling offered to the ERHTEC intervention arm, the regular repeat HIV testing and counseling that both study arms’ participants received throughout follow-up may have heightened HIV risk awareness and risk avoidance behavior among both control and intervention participants to the extent that the relative effect of the ERHTEC counseling intervention was diminished. Moreover, a study effect may have prompted regular (non-intervention) clinic counselors to provide better post-test prevention counseling to control participants, thereby reducing the relative impact of enhanced counseling on intervention participants. The frequency of the study follow-up schedule in itself may have also elicited an unintended risk reduction effect on both intervention and control participants. These hypotheses warrant further research.

Lastly, enhanced counseling may not have been delivered to intervention women as per protocol despite the repeated training and refreshers provided to study counselors during the course of the study. However, qualitative data collected from intervention participants at end of follow-up do not appear to support this possibility as a majority of interviewees perceived the intervention as helpful, supportive and empowering (manuscript in preparation).

We observed a low incidence of HIV over follow-up with a total of four confirmed HIV seroconversions during 1,439 women-years of follow-up. Two occurred in each study arm, and three of them among women enrolled individually. However, the male partner of the woman with whom he enrolled stopped attending follow-up visits and was no longer communicating with her by the 6-month postpartum visit. At or before the time of their seroconversion, all four confirmed HIV-positive women thus reported they were not surprised by their HIV test result as their partner was having additional sexual partners, refused to use condoms, and or had not disclosed his HIV status yet the women knew or suspected them to be infected. These women would have been prime candidates for pre-exposure prophylaxis (PrEP) but PrEP was not part of Uganda’s HIV prevention recommendations at the time of the study. Today, PrEP is recommended by Uganda’s Consolidated Guidelines for the Prevention and Treatment of HIV for HIV-uninfected individuals at high risk of acquiring HIV [49], but implementation of this recommendation remains limited due to restricted access to PrEP drugs in public health facilities throughout Uganda.

While this trial was not powered to compare HIV or STI incidence, the resulting overall HIV incidence rate of 0.186 (95% CI: 0.00–0.52) infections per 100 person-years (PY) of follow-up among all trial participants, as well as the rate of 0.278 (0.00–0.52)/100 WY observed among women participants, are 3.0–3.5 times lower than the respective overall and women incidence rates of 0.66/100 PY (0.53–0.81) and 0.84/100 WY (0.64–1.08) that have been recently reported among nearly 18,000 HIV-negative members of the Rakai Community Cohort Study, an open population-based cohort aged 15 to 49 years in Rakai District, Southern Uganda.[50] In contrast to PRIMAL participants, the Rakai cohort had been the beneficiary of 12 years of sustained combination HIV prevention interventions including HIV counseling and testing, voluntary medical male circumcision, ART for persons living with HIV, as well as promotion of condom use and risk reduction behavior.[50] The PRIMAL rates are also between 4 and 7 times lower than the rate of 1.33/100 PY (0.74–2.40) observed among pregnant and postpartum women receiving a community-based combination HIV prevention intervention in South Africa.[51] Although these three populations may not be directly comparable, our observed rates are also several-fold lower than Uganda’s lowest historical pregnancy and postpartum HIV incidence rates, ranging from 1.3/100PY in postpartum women in 2005[4] to 1.6/100PY in pregnant women in 2013. [52] This low HIV incidence rate is corroborated by a similarly low STI incidence among both intervention and control participants.

Taken together, these results may therefore point to a possible sustained risk-averting effect of repeat HIV and STI testing throughout the breastfeeding period, and this effect may have normalized or reinforced the preventive counselling messages delivered by both, research and regular clinic counselors. In the past few years, an increasing number of publications have reported on the acceptability, feasibility, experiences and cost-effectiveness of repeat HIV testing in late pregnancy.[19–22, 44, 53, 54] This study shows that extended repeat HIV testing and counseling throughout breastfeeding was feasible, acceptable and well received. PRIMAL trial participants were drawn from a large urban public hospital (Mulago) and a rural missionary hospital (St Joseph) that both served the general population and acted as general referral facilities and thus can be assumed to fairly represent the general population of pregnant and lactating mothers in the country. While PrEP may be the prophylaxis of choice for HIV-uninfected women at high risk of acquiring HIV, extended repeat HIV testing and counseling could be a feasible and acceptable complementary way to monitor and reinforce primary HIV acquisition prevention among breastfeeding women at lower risk for HIV. This may warrant serious consideration in light of recent results showing that in pregnant women, the risk of HIV transmission per sex act steadily increases through pregnancy and is highest up to 6 months postpartum.[55]

### Study limitations

Our study has several limitations. Twenty-four percent of study participants were lost before completing follow-up; however, overall, women completed over 80% of their follow-up time and there was no difference between arms. Condom use was assessed through participant recall and therefore subject to potential bias. As discussed above, the frequent study follow-up schedule as well as offering repeat postpartum HIV testing routinely to both intervention and control participants, may have prompted risk reduction behavior among both intervention and control participants, thereby reducing the effect of the intervention. HIV serodiscordance status could not be assessed reliably due to reliance on self-reports by women enrolled individually, and to irregular attendance to follow-up visits by enrolled male partners. STI detection relied on rapid diagnostic tests (RDTs) and were not systematically confirmed by nucleid acid or PCR testing. This may explain the low STI rates we observed relative to those reported among pregnant women in other sub-Saharan countries. [56] Our data however compare to rates reported in the early 2000s in Rakai, except for *T*. *vaginalis*.[57] This may be ascribed to the fact we used the wet mount microscopy technique for detecting *T*. *vaginalis* whose viability and motility are highly dependent on environmental factors.[58] Lastly, as already mentioned, this study was not powered to compare HIV or STI incidences, or to assess interactions between treatment assignments and enrollment status (individuals or couples) or study sites.

## Conclusion

Most PMTCT efforts to date have focused on achieving viremic suppression among HIV-infected mothers. To our knowledge, this is the first randomized controlled study looking at primary prevention of HIV acquisition for up to 24 months postpartum in lactating women in sub-Saharan Africa. Given the substantial HIV acquisition risk breastfeeding mothers continue to be exposed to in sub-Saharan, [4, 55] how to keep HIV-uninfected pregnant women HIV-free throughout pregnancy, delivery and the entire breastfeeding period remains an important research and implementation question that must be answered to achieve elimination of MTCT of HIV. Until an effective HIV vaccine is found, behavioral interventions alone or combined with biomedical/PrEP interventions remain the strongest strategies available to reduce the risk of HIV acquisition among pregnant and postpartum women.

This study did not demonstrate a significant effect of enhanced HIV prevention counseling in increasing condom among HIV-negative pregnant or lactating Ugandan women. We surmise this result may have been due to a study effect on the control group and or to a possible preventive effect of extended repeat HIV testing *per se* on both study arm participants. Although this assumption cannot be directly inferred from our results, the remarkably low HIV incidence rate and STI rates observed among both intervention and control participants may point to a possible sustained preventive effect of extended repeat HIV testing and counseling throughout follow-up. Further research is needed to verify this hypothesis and assess the potential role that extended repeat HIV testing and counseling could play in complementing PrEP for reducing HIV primary and secondary acquisition among pregnant and lactating women at lower risk for STI and HIV.

## Supporting information

S1 Checklist PRIMAL study CONSORT 2010 checklist. (DOC)

S1 Protocol PRIMAL study protocol v5.1 2016. (DOC)

S1 Table Couple HIV counseling and testing (n) among enrolled couples (N), by visit. ^**†**^ N represents the number of women enrolled as couples who presented at a given visit; n represents the fraction of N women who were counselled at a given visit together with their male partner.
* Screening and enrolment; ** Labor and delivery; *** pp = postpartum.
(TIF)

S2 Table Condom use analyses restricted to women who reported no condom use at baseline. (PDF)

S3 Table Sub-analyses of STI incidence over follow-up by study site (Kampala / Kitgum) and enrolment status (individuals / couples). (PDF)

S1 Fig Proportion of intervention and control women reporting they were practicing safer sex in the last 3 months, by visit. Proportion of intervention (E) and control (C) women who reported practicing safer sex through abstinence, condom use and or faithfulness. Women could report more than one safer sex practices. Other practices (counting days, non-penetrative sex) accounted for less than 7% of responses for either group at any visit.
(TIF)

S2 Fig Proportion of consistent or intermittent condom users among intervention and control women in the last 3 months, by visit and study site. ERHTEC-KA / ERHTEC-KI: Intervention group-Kampala / Intervention group-Kitgum.
Control-KA / Control-KI: Control group-Kampala / Control group-Kitgum.
Effect on average change: p = 0.998.
Effect on average follow-up level: p = 0.921.
(TIF)

S3 Fig Proportion of vaginal sex episodes w/ condoms among intervention and control women in the last 3 months, by visit and study site. ERHTEC-KA / ERHTEC-KI: Intervention group-Kampala / Intervention group-Kitgum.
Control-KA / Control-KI: Control group-Kampala / Control group-Kitgum.
Effect on average change: p = 0.648.
Effect on average follow-up level: p = 0.693.
(PDF)

S4 Fig Proportion of condom users at last vaginal sex among intervention and control women in the last 3 months, by visit and study site. ERHTEC-KA / ERHTEC-KI: Intervention group-Kampala / Intervention group-Kitgum.
Control-KA / Control-KI: Control group-Kampala / Control group-Kitgum.
Effect on average change: p = 0.415.
Effect on average follow-up level: p = 0.751.
(TIF)

S5 Fig Proportion of consistent or intermittent condom users among intervention and control women in the last 3 months, by visit and enrolment status. ERHTEC-I: Intervention group-enrolled individually.
ERHTEC-C: Intervention group-enrolled with partner.
Control-I: Control group-enrolled individually.
Control-C: Control group-enrolled with partner.
Effect on average change: p = 0.434.
Effect on average follow-up level: p = 0.370.
(TIF)

S6 Fig Proportion of vaginal sex episodes w/ condoms among intervention and control women in the last 3 months, by visit and enrolment status. ERHTEC-I: Intervention group-enrolled individually.
ERHTEC-C: Intervention group-enrolled with partner.
Control-I: Control group-enrolled individually.
Control-C: Control group-enrolled with partner.
Effect on average change: p = 0.804.
Effect on average follow-up level: p = 0.604.
(TIF)

S7 Fig Proportion of condom users at last vaginal sex among intervention and control women in the last 3 months, by visit and enrolment status. ERHTEC-I: Intervention group-enrolled individually.
ERHTEC-C: Intervention group-enrolled with partner.
Control-I: Control group-enrolled individually.
Control-C: Control group-enrolled with partner.
Effect on average change: p = 0.530.
Effect on average follow-up level: p = 0.290.
(TIF)
