# Supplementary material for: Primary HIV prevention in pregnant and lactating Ugandan women: A randomized trial
Source: PLoS One. 2019 Feb 25;14(2):e0212119. doi: 10.1371/journal.pone.0212119 (PMC6388930; doi:10.1371/journal.pone.0212119)
Supplement: S1 Protocol — (DOC) [file pone.0212119.s002.doc]

**primary hiv prevention in pregnant and lactating ugandan women:**

**a randomized trial**

**(‘PRIMAL’ Study)**

**a University of California San Francisco (ucsf) / Makerere university– johnS hopkins university (MU-JHU) CARE
collaboratiVE study**

**PROTOCOL Version 5.1**

**07 October 2016**

Study title: **Primary HIV Prevention in Pregnant and Lactating Ugandan Women: A Randomized Trial**

Short title: **The ‘PRIMAL’ Study**

Sponsor**: NIH / Eunice Kennedy Shriver National Institute of Child Health & Human Development (NICHD)**

**Grant # 1R01HD070767-01A1**

**UCSF Investigator of Record (Protocol Chair) MU-JHU Care** **Co-Investigator (MU-JHU PI)**

Jaco Homsy, MD, MPH Prof. Elly Katabira, MBChB, MPH

c/o MU-JHU Care MU-JHU Care

Upper Mulago Hill Road Upper Mulago Hill Road

P.O. Box 23491, Kampala, Uganda P.O. Box 23491, Kampala, Uganda

Tel: +256-414-541044, Fax: +256-414-543002 Tel:+256-414-541044, Fax: +256-414-543002

E-mail: [jaco251099@vtx.ch](mailto:jaco251099@vtx.ch) / [jhomsy@psg.ucsf.edu](mailto:jhomsy@psg.ucsf.edu) E-mail: [katabira@imul.com](mailto:katabira@imul.com)

**MU-JHU Care Co-Investigator (Protocol Vice-Chair) AVSI Co-Investigator (Protocol Vice-Chair)**

Zikulah Namukwaya, MBChB, MPH Femke Bannink, MA, PHD cand

MU-JHU Care National Program Coordinator, AVSI Uganda

Upper Mulago Hill Road Plot 1119, Ggaba Road,

P.O. Box 23491, Kampala, Uganda P.O. Box 6785, Kampala, Uganda

Tel: +256-414-541044, Fax: +256-414-543002 Tel:+256-414-501604/5, Fax: +256-414-349547

E-mail: [znamukwaya@mujhu.org](mailto:znamukwaya@mujhu.org) E-mail: [femke.bannink@avsi.org](mailto:femke.bannink@avsi.org)

**NICHD Program Officer NICHD Grants Management Specialist**

Lynne Mofenson, MD Mario Martinez, MPH

Deputy Branch Chief, CRMC/NCMRR Team Leader

Pediatric, Adolescent and Maternal AIDS Branch Grants Management Branch

6100 Executive Boulevard, Room 4B11D, MSC 7510 6100 Executive Blvd, Room 8A07D, MSC 7510

Rockville, MD 20892-7510, USA Bethesda, MD 20892-7510, USA

Telephone: 301-435-6870 /Fax: 301-496-8678 Phone: (301) 402-4078 Fax: (301) 451-5510

Email: [mofensol@exchange.nih.gov](mailto:mofensol@exchange.nih.gov) Email: [martinem@mail.nih.gov](mailto:martinem@mail.nih.gov)

**Protocol Roster/ Site investigators:**

Prof. Mary Glenn Fowler, MD MPH Josaphat Byamugisha, MBChB, MMed, PhD

MU-JHU Care Head, Dept of Obstetrics and Gynecology

Upper Mulago Hill Road Makerere University, Kampala, Uganda

P.O. Box 23491, Kampala, Uganda P.O. Box 7072, Kampala, Uganda

Tel: 256-414-541044, Fax: 256 414 543002 Tel: +256-772-580330; Fax: +256-414-533451

E-mail: [mgfowler@mujhu.org](mailto:mgfowler@mujhu.org) Email: [byamugisha2001@yahoo.com](mailto:byamugisha2001@yahoo.com)

Rachel King, MPH, PhD Lawrence Ojom, MBChB

Director, UCSF-MUSPH META Project Medical Superintendent, St Joseph's Hospital

Plot 30A, York Terrace, Kololo, Kampala, Uganda P.O Box 31, Kitgum, Uganda

Tel: +256-785-304516 / Fax: +256-414-533957 Tel: + 256- 772 611 929 / Fax:+256-414-533957

Email: [rach@vtx.ch](mailto:rach@vtx.ch) / [rlking@psg.ucsf.edu](mailto:rlking@psg.ucsf.edu) Email: [law_ojom@yahoo.co.uk](mailto:law_ojom@yahoo.co.uk)

SOM-REC Number: Ref # 2012-157

UCSF-CHR Number: IRB # 11-08151 – Ref # 055475

**TABLE OF CONTENTS**

[List of abbreviations iv](#__RefHeading___Toc332371389)

[ABSTRACT AND SPECIFIC AIMS 1](#__RefHeading___Toc332371390)

[PROTOCOL SUMMARY 2](#__RefHeading___Toc332371391)

[SCHEMA 4](#__RefHeading___Toc332371392)

[A. INTRODUCTION 5](#__RefHeading___Toc332371394)

[Background and significance 5](#__RefHeading___Toc332371395)

[Study concept 5](#__RefHeading___Toc332371396)

[B. RESEARCH OBJECTIVES AND HYPOTHESES 6](#__RefHeading___Toc332371397)

[Main objectives of the study 6](#__RefHeading___Toc332371398)

[Hypotheses 6](#__RefHeading___Toc332371399)

[Specific objectives 6](#__RefHeading___Toc332371400)

[C. DESIGN AND METHODS 6](#__RefHeading___Toc332371401)

[C.1 Study Design 6](#__RefHeading___Toc332371402)

[C.2 Study setting and populations 7](#__RefHeading___Toc332371403)

[C.3 Intervention components 7](#__RefHeading___Toc332371404)

[C.4 Participants screening and enrolment 8](#__RefHeading___Toc332371405)

[C.4.1 Initial screening 8](#__RefHeading___Toc332371406)

[C.4.2 Inclusion criteria 8](#__RefHeading___Toc332371407)

[C.4.3 Exclusion criteria 8](#__RefHeading___Toc332371408)

[C.4.4 Informed consent 8](#__RefHeading___Toc332371409)

[C.4.5 Enrolment procedures 9](#__RefHeading___Toc332371410)

[C.4.6 Randomization 9](#__RefHeading___Toc332371411)

[C.5 Follow-up 9](#__RefHeading___Toc332371412)

[C.5.1 Retpeat HTC in late pregnancy (>36 weeks) 9](#__RefHeading___Toc332371413)

[C.5.2 Postpartum intervention schedule and assessments 10](#__RefHeading___Toc332371414)

[C.5.3 Sample storage for future use 10](#__RefHeading___Toc332371415)

[C.5.4 Cohort retention methods 10](#__RefHeading___Toc332371416)

[C.5.5 Incident infections 11](#__RefHeading___Toc332371417)

[C.5.6 Study participant compensation and benefits 11](#__RefHeading___Toc332371418)

[C.5.7 Premature study withdrawal 11](#__RefHeading___Toc332371419)

[C.6 Data collection 11](#__RefHeading___Toc332371420)

[C.6.1 Quantitative data 11](#__RefHeading___Toc332371421)

[C.6.2 Qualitative data 11](#__RefHeading___Toc332371422)

[C.7 Measures 12](#__RefHeading___Toc332371423)

[C.7.1 Primary outcome 12](#__RefHeading___Toc332371424)

[C.7.2 Secondary outcomes 12](#__RefHeading___Toc332371425)

[C.7.3 Predictor variables 13](#__RefHeading___Toc332371426)

[C.7.4 Qualitative measurements 13](#__RefHeading___Toc332371427)

[C.7.5 Costs data 13](#__RefHeading___Toc332371428)

[C.8 Statistical considerations and analysis plan 13](#__RefHeading___Toc332371429)

[C.8.1 Preliminary analyses 13](#__RefHeading___Toc332371430)

[C.8.2 Primary analyses 14](#__RefHeading___Toc332371431)

[C.8.3 Secondary analyses 14](#__RefHeading___Toc332371432)

[C.8.4 Cost and cost-effectiveness 14](#__RefHeading___Toc332371433)

[C.8.5 Sample size and power 15](#__RefHeading___Toc332371434)

[C.8.6 Qualitative data analysis 15](#__RefHeading___Toc332371435)

[C.8.7 Data quality and management 15](#__RefHeading___Toc332371436)

[D. HUMAN SUBJECTS PROTECTION 16](#__RefHeading___Toc332371437)

[D.1 Ethical and regulatory considerations 16](#__RefHeading___Toc332371438)

[D.2 Institutional Review Board (IRB) or Independent Ethics Committee (IEC) 16](#__RefHeading___Toc332371439)

[D.3 Informed consent 16](#__RefHeading___Toc332371440)

[D.4 Study completion 16](#__RefHeading___Toc332371441)

[D.5 Publications and dissemination of study findings 17](#__RefHeading___Toc332371442)

[E. TIMELINE 17](#__RefHeading___Toc332371443)

[References 18](#__RefHeading___Toc332371444)

[Appendix A - **Formative Focus Group Discussion points** 20](#__RefHeading___Toc332371445)

[Appendix B - **Counseling Guide Outline** 21](#__RefHeading___Toc332371447)

[Appendix C - **Schedule of Events – Women & Men** 22](#__RefHeading___Toc332371448)

[Appendix D - End of Study Qualitative Intervention Assessments 24](#__RefHeading___Toc332371449)

# List of abbreviations

ANC Antenatal care

ART Antiretroviral therapy

ARV Antiretroviral

AVSI International Service Volunteers Association

ERHTEC Extended HIV repeat testing and enhanced counseling

FGD Focus group discussion

FP Family planning

G/C Gonorrhea / chlamydia

GCP Good Clinical Practices

HTC HIV testing and counseling

ICH International Conference on Harmonization

IEC Independent Ethics Committee

IRB Institutional Review Board

KII Key informant interview

MNCH Maternal, newborn and child health

MTCT Mother-to-child transmission

MU-JHU Makerere University – Johns Hopkins University Research Collaboration

PCR Polymerase chain reaction

PMTCT Prevention of mother-to-child HIV transmission

SEC Science and Ethics Committee

SMS Short messaging system

SOC Standard of care

SOP Standard operating procedure

sSA Sub-Saharan Africa

SOMREC School of Medicine Research and Ethics Committee

STI Sexually transmitted infection

Tv Tricnomonas vaginalis

UCSF University of California San Francisco

UNCST Uganda National Council for Science and Technology

WHO World Health Organization

# ABSTRACT AND SPECIFIC AIMS

The vast majority of the millions of pregnant women who test for HIV every year through prevention of HIV MTCT (PMTCT) programs are HIV-uninfected. Keeping these women uninfected throughout pregnancy and lactation is the first pillar of the WHO global PMTCT strategy. Evidence increasingly shows that HIV-uninfected pregnant women are at continuous risk of HIV acquisition during pregnancy and breastfeeding with HIV incidence rates ranging between 2 and 17 infections per 100 person-years in sub-Saharan Africa. Acquiring HIV during pregnancy or breastfeeding puts mothers at increased risk of adverse health and pregnancy outcomes, and their unborn or breastfeeding babies at higher risk of HIV infection because of the peak viremia that follows incident HIV infection. In addition, pregnancy may increase women’s vulnerability to HIV. For these reasons, WHO recommends that pregnant women who test HIV negative at their first antenatal visit retest in the third trimester of their pregnancy in order to identify acute or incident infection and initiate appropriate PMTCT prophylaxis as early as possible. However, this recommendation has not been widely implemented and its effectiveness has not been assessed. Moreover, the WHO recommendation does not address the risk of incident HIV infection and ensuing vertical transmission during breastfeeding, yet in HIV-uninfected populations in resource-limited settings breastfeeding often lasts up to 2 years.

African women in general and pregnant women in particular are heavily influenced by their male partners when choosing and implementing HIV risk reduction practices. Couples’ HIV testing and counseling (HTC) increases uptake of HIV prevention and care services and is an effective strategy for identifying HIV sero-discordant couples and availing them with all options to prevent horizontal and vertical HIV transmission. Models of couple mobilization and CHCT have been implemented but the effect of these models on sexual risk behavior and HIV transmission has not been systematically evaluated in pregnant women.

In this study, we will test the hypothesis that 1) extended repeat HIV testing and enhanced counseling (ERHTEC) in late pregnancy (>36 weeks of gestational age) and throughout breastfeeding can increase and sustain risk reduction behaviors and prevent incident sexually-transmitted infections (ST)I and HIV infections among HIV-uninfected pregnant women, and 2) that couple HTC can further enhance this effect through improved couple communication and emotional and economic support from male partners. We will conduct a stratified randomized trial in Mulago, Kampala and St Joseph Kitgum hospitals in Uganda. The intervention will consist in providing HIV-negative pregnant women enrolled individually or in couples extended repeat HIV testing and enhanced counseling (ERHTEC) in late pregnancy (>36 weeks) and at 3, 6 12, and 18 months postpartum. ERHTEC will emphasize reinforced risk reduction behavior centered around the concept of ensuring an HIV-free and healthy baby and family. The comparison group will receive repeat standard HTC in late pregnancy and standard counseling during follow-up.

The specific aims of the study are:

Aim 1: to assess the effect of ERHTEC on sexual risk behavior and STI and HIV acquisition in HIV-uninfected pregnant and lactating women enrolled individually

Aim 2: to assess the effect of ERHTEC on sexual risk behavior and the incidence of STIs and HIV in uninfected pregnant and lactating women enrolled with their partner, and

Aim 3: to assess the costs and estimate the cost-effectiveness of the intervention.

To accomplish these objectives, we will enroll 410 HIV-negative pregnant women individually and 410 HIV-negative pregnant women with their partners from Mulago and St Joseph Kitgum Hospitals ANC clinics. Individual women and couples will be randomized 1:1 to receive the intervention or the standard of care as described above. Participants will be followed up until 24 months postpartum or 6 weeks after the end of breastfeeding, whichever occurs first. Frequency of unprotected sex and incidences of HIV, other STI, and unintended recurrent pregnancies will be compared in intervention and comparison arms. Quantitative and qualitative data on individual and relational mediators of risk behaviors will be collected over follow-up including couple communication, partner economic and emotional support, vertical transmission of HIV and reproductive intentions. Possible associations of individual and dyadic variables with the primary and secondary outcomes will be analyzed and the cost-effectiveness of the intervention will be estimated.

The findings of this study will inform national policy and guidelines about the effect of retesting HIV-negative pregnant and lactating women and their partners during pregnancy and breastfeeding on primary HIV prevention.

# PROTOCOL SUMMARY

**Background**

**Design**

This study consists in a stratified randomized trial, a qualitative formative component at baseline, a qualitative assessment at end of follow-up, and a cost-effectiveness analysis.

Formative research qualitative component**:** Focus Group Discussions (FGDs) will be conducted at baseline to explore knowledge, beliefs, attitudes, challenges and expectations about primary HIV prevention among pregnant and lactating women and their partners.

Stratified randomized trial component**:** A stratified randomized trial will be conducted to compare an extended HIV repeat testing and enhanced counselling (ERHTEC) intervention aimed at preventing HIV acquisition in pregnant and lactating women to the current Ministry of Health recommendation to retest and counsel HIV-uninfected women in late pregnancy (>36 weeks).

End of follow-up qualitative assessment: FGDs and key informant interviews (KIIs) will be conducted at end of follow-up to assess the acceptability and feasibility, barriers and facilitators of the ERHTEC intervention

Additional research components:

Cost-effectiveness of the ERHTEC intervention

**Population**

Study population for the formative phase: HIV-negative pregnant women and HIV-negative postpartum mothers (FGD 1); partners with any HIV status of HIV-negative pregnant women and HIV-negative post-partum mothers (FGD 2); doctors/clinical officers, nurses/midwives, counsellors, and peers (FGD 3).

Study population for the randomized trial phase: HIV-uninfected pregnant women from the Antenatal Clinics (ANC) of Mulago National Referral Hospital in Kampala, Uganda and St Joseph Hospital* in Kitgum, Uganda.

**Kitgum District Hospital will also be included if participant accrual from St Joseph Hospital is insufficient.*

Study population for the end of follow-up qualitative assessment: FGDs will be conducted with study staff and trial participants (women and men). KIIs will be conducted with HIV-negative mothers, seroconverted mothers, partners of seroconverted mothers and HIV-infected partners.

**Sample size**

Sample size for the formative research phase: Six FGDs, 3 in each site. Each FGD will include 8-10 members

Sample size for the randomized trial phase**:** 1,230 participants (205 women and 205 couples per study arm equally distributed among the urban (Kampala) and rural (Kitgum) sites.

Sample size for the end of follow-up qualitative assessment: Fifty-five KIIs (20 in Kitgum, 35 in Kampala) and 9 FGDs (3 in Kitgum, 6 in Kampala). Each FGD will include 8-10 members.

**Main study goal**

The study goal is to assess the effectiveness of a counseling and testing intervention aimed at preventing primary HIV acquisition in pregnant and lactating women.

Specific objective of the formative phase:

To explore factors affecting awareness, beliefs and attitudes about primary HIV prevention among pregnant and lactating women and their partners.

Specific aims of the randomized trial phase:

- Aim 1: to assess the effect of ERHTEC on sexual risk behavior and STI and HIV acquisition in HIV-uninfected pregnant and lactating women enrolled individually
- Aim 2: to assess the effect of ERHTEC on sexual risk behavior and the incidence of STIs and HIV in HIV-uninfected pregnant and lactating women enrolled with their partner
- Aim 3: to assess the costs and estimate the cost-effectiveness of the intervention.

Specific objective of the end of follow-up qualitative assessment:

To document qualitatively the acceptability and feasibility of the intervention

**Duration**

The qualitative formative phase is planned to last 3 months. The randomized trial phase is planned to last 3 years counting 6-9 months for enrolment and 27-30 months of follow-up. The end-of-follow-up assessment phase is expected to last 6 months

**Intervention**

The intervention is an extended HIV retesting and enhanced counselling package to prevent HIV acquisition in HIV-uninfected pregnant and lactating women

**Study sites**

Mulago National Referral Hospital, Kampala, Uganda and St Joseph Hospital* in Kitgum, Uganda.

*Kitgum District Hospital will also be included if trial participant accrual from St Joseph Hospital is insufficient.

**Study outcomes**

Formative research outcomes:

Perceptions, attitudes, expectations and potential barriers about men involvement in (MNCH care, and an ERHTEC intervention.

Primary outcomes for the randomized trial

Comparison of unprotected sex frequency, and HIV and STI incidence rates among intervention (ERHTEC) and control (standard WHO recommendation) arms .

Cost-effectiveness of the ERHTEC intervention compared to standard of care

Secondary outcomes for the randomized trial

Comparison of unintended recurrent pregnancy rates across study arms

Associations of individual and dyadic variables with the primary and secondary outcomes

Maternal and infant mortality rates

Additional study outcomes

Trial participants’ and staff’s experiences and perceptions about the intervention

# SCHEMA

Mulago National St Joseph Hospital /

Referral Hospital (*Kitgum District Hospital**)

Kampala Kitgum

Follow - up up to 24 months** postpartum

Arm I-1* - Standard repeat HIV testing and counseling (HTC) in late pregnancy

Individual women (N=205)

**Pregnant women in ANC clinics**

**Formative research**: Focus group discussions on knowledge, beliefs, attitudes, challenges and expectations of pregnant and postpartum women, partners and staff about primary HIV prevention

Arm I-2* - Extended repeat HIV retesting and enhanced counselling (ERHTEC)

Individual women (N=205)

Results to inform intervention

Arm C-1* - Standard repeat HIV testing and counseling (HTC) in late pregnancy

Couples (N=205 women and 205 men)

**Stratified randomized trial**

Participant screening, enrolment & randomization (N=1,230 HIV-negative women)

** Kitgum District Hospital will be only used if trial participant
accrual from St Joseph Hospital is insufficient*

Arm C-2* - Extended repeat HIV retesting and enhanced counselling (ERHTEC)

Couples (N=205 women and 205 men)

Primary outcomes**:

- Rates of unprotected sex, and HIV and STI incidences

- Costs and cost-effectiveness of the intervention

Secondary outcomes**:

- Rates of unintended recurrent pregnancies

- Associations of individual and dyadic variables with primary and secondary outcomes

- Maternal and infant mortality rates

- MTCT of HIV among women acquiring HIV during follow-up

Additional outcomes:

- Acceptability/feasibility, and barriers/facilitators of the intervention

** I-1 = individual control arm / I-2 = individual intervention arm / C-1 = couple control arm / C-2 = couple intervention arm*

*** Both primary and secondary outcomes will be assessed at the end of follow-up, defined as 24 months postpartum or 6 weeks after the end of breastfeeding, whichever occurs first*

# A. INTRODUCTION

## Background and significance

Sub-Saharan Africa (sSA) accounts for 90% of new mother-to-child HIV transmission (MTCT) infections and more than 15% of all new HIV infections worldwide[^1^](#_ENREF_1). The WHO Global Strategy to achieve the elimination of mother-to-child HIV transmission of HIV emphasizes primary as well as secondary HIV transmission prevention in women of childbearing age[^2^](#_ENREF_2). Evidence increasingly shows that HIV-negative pregnant and breastfeeding women are at continuous risk of HIV acquisition in sSA with incidence rates ranging between 2 and 17 infections per 100 person-years[^3-14^](#_ENREF_3). Pregnancy may increase women’s susceptibility to HIV and HIV acquisition during pregnancy or breastfeeding carries a highly increased risk of MTCT and may carry an increased risk of adverse pregnancy and birth outcomes. For all these reasons, it is not only imperative to retest women regularly during pregnancy and breastfeeding to ensure prompt PMTCT intervention in case of maternal HIV acquisition, but it is equally important to prevent HIV acquisition in uninfected mothers and ensure that they adopt risk reduction behaviors to remain HIV-free and protect their baby.

WHO recommends that in settings with generalized epidemics, pregnant women who test HIV-negative on their first ANC visit be retested in late pregnancy (>36 weeks) or at labor or immediately after delivery[^16^](#_ENREF_16). HIV post-test counselling provides the opportunity to both, address high-risk behavior and reinforce risk-reduction choices and practices. Evidence shows that preventing HIV infections is far more cost-effective than treating them[^17^](#_ENREF_17) and that the majority of new infections in sSA are occurring in married, cohabiting HIV sero-discordant couples who do not know their HIV status and are unaware of their risk[^18-26^](#_ENREF_18). Yet, there is little if any evidence-based strategy for the primary prevention of HIV prevention in pregnant women. Such evidence is particularly needed in resource-limited settings where the bulk of MTCT of HIV occurs.

For many African women, making and implementing risk reduction or reproductive health decisions are heavily dependent on their partner’s consent. Couple HTC is effective in reducing sexual risk, especially for serodiscordant couples, and in increasing PMTCT uptake. To date, couple HTC for PMTCT remains largely a missed opportunity[^27-31^](#_ENREF_27) as few PMTCT programs in sSA have succeeded in mobilizing and testing couples, and fewer still fully address the prevention needs of HIV-uninfected pregnant women[^32^](#_ENREF_32). Yet PMTCT programs are an important entry point for the primary prevention of HIV in pregnant women and their families.

## Study concept

To our knowledge, this study will be the first randomized trial to assess an intervention promoting the primary prevention of HIV in pregnant and lactating women individually or in couples. The intervention consists in delivering extended HIV repeat testing coupled with enhanced counseling (ERHTEC) and community support throughout the lactation period with the aim of motivating and empowering women and couples to keep their baby and family free of HIV.

The enhanced counseling intervention will draw on the natural desire of pregnant and breastfeeding women and couples to keep HIV away or at bay from their baby and family. Through this approach, counselors will promote three key mother, newborn and child health (MNCH) and HIV prevention behaviors: safe sex, optimal infant feeding and care, and planning any desired subsequent pregnancy in a way that ensures optimal health of the new baby and its mother.

Counseling and education messages and guidelines need to be tailored to individual women and couples at key stages of the pregnancy and post-natal periods. In many sSA settings, the resumption of sexual activity after delivery is a critical moment for HIV prevention interventions[^33^](#_ENREF_33). The ERHTEC intervention will connect individual mothers and couples to family support groups including peer mothers and fathers who will be trained as mentors and lay counselors for primary HIV prevention and to support study participants to adhere to sound infant feeding practices, child care and immunization schedules, to make informed decisions regarding family planning, and to access prompt PMTCT prophylaxis and HIV care and treatment in case of incident infection of the baby and/or the mother. Couples found to be or become serodiscordant over follow-up will be referred to receive all pre-exposure prophylaxis options recommended at the time of the study, including male medical circumcision and tenofovir-based chemoprophylaxis or vaginal gel if recommended.

The study will take place in Mulago and St Joseph Kitgum hospitals that have implemented effective couple mobilization and couple HTC strategies for PMTCT that have resulted in high rates of male partner participation in antenatal, intra- and post-partum MCH care. This level of couple participation will allow us to better understand the role male partners could play in promoting and sustaining primary HIV prevention

# B. RESEARCH OBJECTIVES AND HYPOTHESES

**Main objectives of the study**

The main goal of this study is to assess the effectiveness of a behavioral intervention aimed at preventing primary acquisition of HIV in pregnant and lactating women enrolled individually or in couples in Uganda.

**Hypotheses**

In this study, we will test the hypotheses that:

1) extended repeat HIV testing and enhanced counseling (ERHTEC) during late pregnancy (>36 weeks) and breastfeeding can increase and sustain risk reduction behaviors and prevent incident STI and HIV infections among HIV-uninfected pregnant women, and

2) that couple HTC can further enhance this effect through improved couple communication and emotional and economic support from male partners.

**Specific objectives**

Formative phase:

To explore factors affecting awareness, beliefs and attitudes about primary HIV prevention in pregnant and lactating women and their partners.

Randomized trial phase:

To assess the effect of the intervention on sexual risk behavior and STI and HIV acquisition in HIV-uninfected pregnant and lactating women enrolled individually (Aim 1) or with their partner (Aim 2).

End of follow-up assessments:

To assess the acceptability and feasibility of the intervention.

To assess the costs and estimate the cost-effectiveness of the intervention.

**C. DESIGN AND METHODS**

**C.1 Study Design**

We will conduct a stratified randomized trial of 820 HIV-negative pregnant women and 410 male partners in Mulago Hospital in Kampala and St Joseph District Hospital in Kitgum, Northern Uganda: in each site 205 women enrolled individually and 205 couples will be randomized to be retested and counseled either in late pregnancy (>36 weeks) only as per the WHO/MOH recommendation, or to receive the ERHTEC intervention throughout pregnancy and breastfeeding as follows:

**PREGNANT WOMEN PRESENTING AT ANC CLINIC**

**WITHOUT PARTNER**

**IN COUPLES**

**Fig. 1**

**FIRST ANC VISIT**

**410 HIV-negative pregnant women enrolled WITHOUT partner**

**410 HIV-negative pregnant women enrolled WITH their 410 partners**

**RANDOMIZATION**

**205 women receive repeat HTC in late pregnancy**

**205 women receive ERHTEC in late pregnancy + at 3,6,12, & 18 mos post-partum**

**205 couples receive repeat HTC in late pregnancy**

**205 couples receive ERHTEC in late pregnancy + at 3,6,12, & 18 mos post-partum^✝^**

**Arm I*-1**

**Arm I*-2**

**Arm C*-1**

**Arm C*-2**

**** I = Individual; C = Couple***

Follow-up will end at 24 months postpartum or 6 weeks after the end of breastfeeding, whichever occurs first. Quantitative data will be collected on participants’ HIV and STI status, socio-demographics characteristics, risk behaviors and perceptions, as well as relational factors and reproductive history and intentions. The primary outcomes will be the frequency of unprotected sex and the incidences of HIV and STI over follow-up. Self-reports of unprotected sex by women participants will be validated by testing women for the presence of semen biomarkers (Y chromosome) in vaginal swabs[^36^](#_ENREF_36). We will also collect qualitative data at baseline and at the end of follow-up on the knowledge, perceptions and experiences of study participants and providers on the challenges of retesting, partner’s involvement and primary HIV prevention.

**C.2 Study setting and populations**

**C.2.1 Setting**

The study will take place at Mulago Hospital in Kampala and St Joseph Kitgum Hospital in Kitgum, Northern Uganda. If recruitment at St Joseph Hospital is not sufficient, we will recruit patients from Kitgum District Hospital as well which is co-located in Kitgum town. All three hospitals have established PMTCT programs and see a substantial number of pregnant women presenting with their partners in the ANC Clinics. Mulago Hospital receives more than 30,000 pregnant women a year in three separate ANC clinics and has housed several landmark clinical trials in collaboration with the Makerere University-Johns Hopkins University Research Collaboration (MU-JHU) which supports the PMTCT program. St Joseph Hospital’s PMTCT program in Northern Uganda has been supported by AVSI Foundation since 2002. All ANC clinics provide comprehensive PMTCT services including couple HTC, and all three hospitals provide adult and pediatric ART, ARV prophylaxis for mothers not eligible for ART and their baby, early infant HIV diagnosis, improved obstetrical practices and infant feeding support as part of the standard of care.

**C.2.2 Study populations**

Study population for the formative phase: HIV-negative pregnant women and HIV-negative postpartum mothers (FGD 1); partners with any HIV status of HIV-negative pregnant women and HIV-negative post-partum mothers (FGD 2); doctors/clinical officers, nurses/midwives, counselors, and peers (FGD 3). Each FGD will involve 8-10 participants.

Study population for the randomized trial phase: 1,230 HIV-uninfected pregnant women from the Antenatal Clinics (ANC) of Mulago National Referral Hospital in Kampala and St Joseph Hospital/Kitgum District Hospital* in Kitgum, Uganda.

**Kitgum District Hospital will be used only if trial participant accrual from St Joseph Hospital is insufficient.*

Study population for the end of follow-up qualitative assessment: Study staff (health providers, ~10 per site) and women and men trial participants including seroconverted individuals and couples, if any (20 per site).

**C.3 Intervention components**

**C.3.1 Formative research.** Qualitative data on pregnant women’s knowledge, beliefs and attitudes towards primary HIV prevention will be collected through focus group discussions with HIV-negative women. In each site, a group of women presenting in ANC clinics individually, a group of women presenting with their partner and a group of women’s partners will be invited to participate in separate FGDs to inform the ERHTEC intervention. The discussions will focus on participants' awareness, understanding, beliefs, attitudes, practices, challenges and opportunities towards primary HIV prevention (see outline of FGDs in Appendix A).

**C.3.2 Counseling guide.** We will adapt the existing MOH and CDC HIV counseling guides for individuals and couple counseling to the specific context of primary HIV prevention for pregnant and lactating women and their partners (See Appendix B for an outline of the PRIMAL counseling guide).

The PRIMAL counseling guide will be pre-tested by two counselors at each site with HIV-negative pregnant and lactating women presenting individually or with their partner. Pretesting counselors will take detailed notes of issues encountered during the pre-test. Women counseled for pretesting will be given retesting appointments and followed up for the duration of the study but will not be enrolled in the study. The guide will be designed for trained counselors and adapted for use by lay counselors including peer mentors and field officers.

**C.3.3 Training of study staff.** MCH staff from Mulago and St Joseph Kitgum Hospitals and community support groups will be sensitized to the study objectives, and technical staff including counselors, lab technicians, research assistants and study coordinators will be trained in good clinical practices (GCP) and human subject protection (HSP) (see Section D. below), and use of the study protocol, standard operating procedures (SOPs) and case record forms (CRFs). Study counselors will also be trained in depth in the use of the counseling guide and in performing rapid HIV, STI and pregnancy testing. All trained staff will be mentored on a regular basis by on-site investigators, counseling supervisors and study coordinators. Field officers will be trained on home visit procedures and ways to maintain confidentiality and reduce stigma associated with study participation. They will also be sensitized to the risk of attrition among women and couples.

**C.3.4 Training of peers and family support groups.** Trained study counselors will in turn train peer counselors in the ERHTEC intervention. Family and women support groups linked to both St Joseph and Mulago Hospitals PMTCT programs will also be sensitized about the study with a focus on helping to retain participants in the study and reinforcing prevention messages. These groups have been instrumental in increasing couple attendance at the ANC clinics and adherence to PMTCT and HIV care and treatment interventions in both sites. We expect they will be equally helpful in keeping the communities informed about the study and in supporting enrolled women and couples to attend study visits.

**C.3.5 Men friendly spaces for couple HTC.** One of the major obstacles to partner involvement in MCH and PMTCT in sSA is the lack of men-friendly areas in and/or around the ANC, labor and delivery and post-natal/MCH units. In both St Joseph and Mulago ANC clinics, we will identify or create spaces suited for couple waiting and counseling and for men waiting while their wife/partner is undergoing ante- or postnatal examinations or is in labor. Men should be invited to watch their baby being examined, weighted and immunized. We will also try and engage community leaders in this process to showcase couple participation in the study. These measures aim to attract couples and to increase the clinics capacity to attend to couples.

**C.3.6 Qualitative assessment of the intervention.** Key informant interviews will be conducted with trial participants to assess the acceptability and feasibility of the ERHTEC intervention. Women and men with different behavioral and biological outcomes will be purposively selected in each site according to the study outcomes. FGDs will also be carried out with study staff to assess the acceptability and feasibility of the intervention. FGDs and KIIs will focus on challenges, opportunities and scalability of the intervention (see FGDs and KIIs guide outlines in Appendix D).

## C.4 Participants screening and enrolment

### C.4.1 Initial screening

Participants will be recruited from Mulago and St Joseph ANC clinics. Midwives/counselors or research assistants will approach pregnant women who have tested HIV-negative and determine whether the women came and intend to continue attending as an individual or with their partner. The staff will briefly inform individual women or couples verbally about the study and its requirements and hand them a printed sheet with the same information. Women and couples agreeable to screening for participation will be referred to the study team. During screening, a study clinician, counselor or research assistant will first assess the woman’s provisional eligibility criteria. If the provisional eligibility criteria are met, women will be asked to provide written consent. Once consented, index women will be retested for HIV and if confirmed HIV-negative, will be assessed for final eligibility. At this point, partners of eligible women intending to join the study as couples will be assessed for eligibility and consented if eligible. Enrolled women/couples will be given an appointment to return within the next 2 weeks to complete baseline exams, tests and procedures. All discussions, interviews and examinations will be done in the appropriate language of participants.

| C.4.2 Inclusion criteria | | ALL |
| --- | --- | --- |
| Women | - Age: 15-49 years - Confirmed pregnancy through clinical assessment or pregnancy test - Documented HIV-negative sero-status at the time of screening - Intention to breastfeed | a) Absence of serious medical condition  b) Willingness to be tested and counseled for HIV and other STIs  c) Residing within a 30 km radius around the study clinic and not planning on moving (*to be checked using place of origin, date of arrival and residence of relatives to reduce the possibility of selective attrition*);  d) Agreeing to come the study clinic for scheduled appointments and to be called by phone or visited at home as needed during follow up  e) Agreeing to provide written informed consent |
| Partners | - Age: >15 years - Recognized by an eligible pregnant woman as her current husband/partner for at least 6 months |

### C.4.3 Exclusion criteria

Women and their partners will be excluded for the following reason:

- Inability to provide informed consent
- Concurrent participation in a randomized trial or research study
- Social or other circumstances which would hinder follow-up during the study in the opinion of the site investigator

### C.4.4 Informed consent

Participants will be asked to provide written consent for the collection of blood, urine and vaginal/urethral swabs for HIV and STI testing, collection of personal data through questionnaires, group discussions and key informant interviews at baseline and follow-up visits, data abstraction from their medical records, and future use of biological specimens obtained during the study. Study counselors will conduct the consent discussion in the appropriate language and a translator will be used if necessary. Both members of couples will be consented together unless either partner opts to be consented individually. Printed consent forms translated in the language of participants and approved by the UCSF Committee for Human Research (CHR), Makerere University School of Medicine Research and Ethics Committee (SOMREC), and the Uganda National Council for Science and Technology (UNCST) will be used to describe the study to potential participants. Following the consent discussion, participants will be asked to sign the consent forms. Participants unable to read or write will use fingerprints in lieu of signature, and a signature will be obtained from a witness to confirm the consent process.

### C.4.5 Enrolment procedures

Consented women and men will be retested for HIV by rapid test. Women with an HIV-positive or indeterminate result will be ineligible for the study and referred back to the PMTCT clinic. Only women with a confirmed HIV-negative result will be enrolled either alone or with their partner, independent of the partner’s HIV serostatus. All newly enrolled participants will be counseled per the study counseling protocol. Both members of couples will be counseled together unless either partner opts to be counseled individually. In case of HIV serodiscordance, the infected partner will be bled after counseling for CD4 testing and referred to the HIV clinic for registration and ART eligibility assessment, if not already done. All participants will undergo a standard clinical examination by the study clinician. All women will be bled for syphilis testing and hemoglobin measurement, and a vaginal swab will be collected for gonorrhea/ chlamydia (G/C) and Trichomonas vaginalis (Tv) testing and to test for the presence of semen (Y chromosome). Men will also be tested for syphilis and a urine sample or urethral swab will be obtained for G/C and Tv testing. All enrolled women and partners will be asked to answer a baseline demographics and behavioral questionnaire about their socio-economic status (SES), sexual and relational behaviors, and reproductive history and intentions, including family planning. All interviews will be conducted in the language of participants. Questionnaires will be translated and back-translated to ensure consistency, accuracy and clarity of the questions.

### C.4.6 Randomization

There are four study arms:

| Individual Arm 1 (Arm I-1) | HIV-negative pregnant women enrolling **without** their partner and who will receive standard HIV retesting and counseling in late pregnancy only |
| --- | --- |
| Individual Arm 2 (Arm I-2) | HIV-negative pregnant women enrolling **without** their partner and who will receive the ERHTEC intervention in late pregnancy AND throughout breastfeeding as described below (C.5 Follow-up) |
| Couple Arm 1  (Arm C-1) | HIV-negative pregnant women enrolling **with** their partner and who will receive standard HIV retesting and counseling in late pregnancy only |
| Couple Arm 2 (Arm C-2) | HIV-negative pregnant women enrolling **with** their partner and who will receive the ERHTEC intervention in late pregnancy AND throughout breastfeeding as described below (C.5 Follow-up) |

Enrolled women and couples will be randomized to either the control (repeat HTC in late pregnancy [>36 weeks] only), or to the ERHTEC intervention. Prior to the study onset, four sets of randomized, sequentially numbered lists of participants identification numbers (IDs) will be prepared by the statistician at UCSF. The randomization lists will be computer-generated using random-sized block groups and will include consecutive intervention numbers with corresponding random intervention assignments. Randomization lists will be encrypted in password-protected files, backed up on computer and the UCSF server and emailed to the MU-JHU Data Center in Kampala. Sealed copies of the original randomization list and documentation of the procedure used to generate the lists will be stored in the project administrative offices in San Francisco and Kampala. Each enrolment number will be preceded with a code letter indicating the trial group (“I” for individual, “F” for the female partner of a couple, “M” for the male partner of a couple) and followed by a randomization allocation code (“E” for ERHTEC intervention, “C” for control) and a site code (“KA” for Kampala, “KI” for Kitgum).. At randomization, MU-JHU Data Center will issue randomization numbers to the study coordinators based on the order of enrollment at each site and on the participants’ group allocation (individual or couple).

## C.5 Follow-up

### C.5.1 Repeat HTC in late pregnancy (>36 weeks)

All women will be given an appointment to be retested and counseled for HIV in late pregnancy as per WHO and Uganda MOH recommendations. Women enrolled at or before the 16^th^ gestational week will be given an appointment for ERHTEC or repeat HTC at 28 weeks of gestation. Women enrolled after the 16^th^ up to the 32^nd^ gestational week will be given an appointment for ERHTEC or repeat HTC at 36 weeks of gestation, or at the first signs of labor, whichever comes first. Women enrolled between the 32^nd^ and 36^th^ gestational week will be given an appointment for ERHTEC or repeat HTC 6 weeks later or at the first signs of labor, whichever comes first. Women enrolled after the 36^th^ gestational week will be given an appointment for repeat HTC at the 6-week post-partum visit to coincide with the first routine immunization visit. In the event that a pregnant woman or couple does not return on the scheduled appointment but presents for delivery, she/they will be retested immediately before or after delivery as per the WHO recommendation. At the late pregnancy retesting visit, all women and men randomized to the intervention arms (Individual and Couple Arms ‘2’) will receive the ERHTEC intervention while women and men randomized to the control arms (Individual and Couple Arms ‘1’) will receive standard HIV retesting and counseling. In addition, both intervention and control women will be bled for syphilis testing and hemoglobin measurement and a vaginal swab will be collected for testing for Gonorrhea/ Chlamydia (G/C), Trichomonas vaginalis (Tv) and the presence of semen (Y chromosome). Symptomatic men will be bled for syphilis testing and asked for a urine sample or urethral swab for G/C and Tv testing. All enrolled women and couples will be asked to answer a follow-up behavioral questionnaire about their sexual and relational behaviors, and reproductive intentions, including family planning. All interviews will be conducted in the language of the participants.

**C.5.2 Postpartum intervention schedule and assessments**

After the late pregnancy visit, all women and couples in the intervention arm will be asked to return at 6 weeks and 3 months postpartum and every 3 months thereafter until the end of follow-up at 24 months postpartum or 6 weeks after the end of breastfeeding, whichever occurs first. Women and couples in the control arm will be asked to return at 3 and 6 months postpartum and every 6 months thereafter until the end of follow-up at 24 months postpartum or 6 weeks after the end of breastfeeding, whichever occurs first.

Participants randomized to the intervention arms (Arms I-2 & C-2) will receive enhanced counseling at every visit and will be retested for HIV at 3, 6, 12, 18 and 24 months postpartum (or at the end of follow-up, whichever occurs first). At these visits, both intervention and control women and men participants will undergo a physical examination and will be asked to answer individually a follow-up behavioral questionnaire about their sexual behaviors, relational and reproductive intentions, including family planning. At these same visits, all women will be bled for syphilis and HIV rapid testing and two vaginal swabs will be collected, one for G/C, and Tv testing and the other for Y chromosome detection while a blood or urine sample or urethral swab will be obtained from symptomatic men for syphilis, G/C and Tv testing [*See Schedule of Events, Appendix C*].

At the end of follow-up, all study participants irrespective of their arm assignment will undergo a physical examination, will be asked to answer individually an end-of-study questionnaire and will be bled for syphilis and HIV rapid testing and a vaginal or urethral swab will be collected for G/C and Tv testing. A vaginal swab will also be collected from women to test for the presence of Y chromosomes.

**C.5.3 Sample storage for future use**

At the end of the study, excess blood, urine and vaginal/urethral swabs samples will be stored in -70^o^C freezers at MU-JHU Care facilities in Mulago or at UVRI CDC laboratory in Entebbe (depending on space availability) for future testing. Tests will be done at either MU-JHU or UVRI laboratory. They may include tests to confirm previous results, to determine if participants had any sexually transmitted infections (STI) or any unprotected sex between assessment visits or to assess for the presence of STIs not tested for during the study such as HSV-2. Tests may also be done to know when exactly seroconverters became infected with HIV or STIs and to assess immunological parameters such as CD4 counts. Test results relevant to participants’ health and medical care will be given to participants and/or their doctor.

Investigators interested in using left over samples will need to submit a request to the PI who will set up an independent panel to review the scientific proposals and make recommendations to the PI before he gives his approval. The PI or any investigator requesting to use the stored samples will be asked to submit a proposal and obtain approval from the Uganda and US IRBs before using the samples. The left over samples will be stored for a minimum of two (2) years after the termination of the study. The specimen will remain the property of the PI or designee for as long as they will be stored.

**C.5.4 Cohort retention methods**

Our study power calculations account for a rate of attrition as high as 50% to ensure sufficient power for the primary endpoint comparisons[[1]](#footnote-2). Given that such a high rate carries a risk of bias, we have put in place strategies to minimize losses to follow-up as follows:

- Participants in the urban or rural site will be carefully assessed at enrolment for their mobility in order to pre-empt selective attrition
- All women and couples consenting to participate will have agreed to home visits by a study field officer.
- At the time of enrollment the field officer will make a home visit and complete a tracking locator form.
- Study retention will be monitored monthly.
- To maximize retention over 24 months of follow-up, study visits have been scheduled to coincide as much as possible with the standard ante-natal or post-natal immunization schedule and additional retention visits have been inserted so that all participants will be on a regular quarterly visit schedule.
- Every participant will be given a free calendar with visits dates marked to help them remember their appointments.
- Trained counselors will maintain regular contact with participants through calls and SMS messages while respecting participants’ schedule and privacy.
- Family and women support groups linked to both Kitgum and Mulago PMTCT programs will also help retain participants in the study by reinforcing prevention messages.
- If a participant misses a follow-up visit, a field officer or a study peer will follow up by phoning as soon as possible to reschedule the visit.
- When needed, the field officer or peer will make a follow-up visit either at the participant’s home or at an agreed upon location.

**C.5.5 Incident infections**

All participants with an incident STI or HIV infection during follow-up will receive treatment as part of standard of care provided by the hospitals and in accordance with Uganda’s National STI Treatment Guidelines[^42^](#_ENREF_42). These guidelines include partner notification and treatment, thus both enrolled and unenrolled partners of participants found to be infected during follow-up will be notified and treated when possible. Participants infected with syphilis and their partners will be treated with benzathine penicillin 2.4 MU IM in a single dose. Pregnant women infected with G/C or T. vaginalis and their partners will be treated with erythromycin 500mg qid for 7 days plus cotrimoxazole 2.4g bid for 3 days and metronidazole 2g single dose (no cotrimoxazole or metronidazole will be given in the first trimester or after 36 weeks of pregnancy). Non-pregnant lactating women and their partners infected with G/C or T. vaginalis will be treated with ciprofloxacin 500mg single dose and metronidazole 2g single dose plus doxycycline100mg bid for 7 days . In case of incident HIV infection in a participant, counselors will emphasize both primary & positive prevention, and a study clinician will obtain a blood sample for CD4 testing and refer the infected person(s) to Mulago or St Joseph Hospitals HIV clinic for ART eligibility assessment. All HIV seroconverters will receive cotrimoxazole prophylaxis and lifetime ART if eligible. Infected pregnant women and breastfeeding women ineligible for ART and their babies will receive PMTCT prophylaxis or treatment as per national guidelines[^32^](#_ENREF_32). All newly HIV-exposed infants (babies born to and/or breastfeeding from an HIV-seroconverted mother) will receive ARV prophylaxis as per national guidelines. HIV-exposed babies >6 weeks of age will be tested by HIV DNA polymerase chain reaction (PCR) assay and if infected, will be treated with triple ART at the hospital HIV clinic. Uninfected infants will be retested 6 weeks after complete cessation of breastfeeding and treated with triple ART if infected.

**C.5.6 Study participant compensation and benefits**

Mulago and Kitgum participants will be reimbursed 10,000 Ugandan Shillings and 5,000 Ugandan Shillings respectively for their time and transport to and from the clinic for each study visit that is not part of routinely scheduled visits for ante-natal, delivery or post-partum care. Participants will also be provided lunch when they will have to stay in the clinic beyond lunch time. Participants who run into hardship and have serious difficulties coming to any given visit will be dealt with on an individual basis. All participants will receive the standard of care and treatment available at the hospitals for obstetrical or medical problem at enrolment or during follow-up. Participants testing HIV-positive will receive cotrimoxazole and ARV prophylaxis for PMTCT as well as HIV/AIDS care and treatment from the hospital HIV clinic at no cost, including ART if found eligible. Provision of any care and treatment will not be contingent upon participating in or withdrawing from the study at any time.

**C.5.7 Premature study withdrawal**

Study participants will be withdrawn before the end of the follow-up period if any of the following event occurs: 1) movement out of study area for >90 consecutive days, 2) failure to attend to or to be located for >2 consecutive scheduled visits, 3) withdrawal of informed consent, 4) inability to adhere to study schedule and procedures, or 5) death of the participant. Women/couples who experience an abortion, still birth, a neonatal or infant death will be censored from follow-up but will continue to be seen by the study staff and receive care from the study clinic for 6 months following the termination of their pregnancy or the death of their child.

**C.6 Data collection**

Quantitative data will be collected from all participants at both study sites until 24 months postpartum or 6 weeks after the end of breastfeeding, whichever comes first. Qualitative data will be collected at specific time points described in C.6.2.

**C.6.1 Quantitative data**

Data on outcomes of interests and on the individual and relational mediators of these outcomes will be collected at different time points over follow-up as outlined in Table 1 below.

**C.6.2 Qualitative data**

Qualitative data collection will be integral to the assessment of risk behavior and will provide insights into the feasibility and acceptability of the intervention. Focus group discussions (FGDs) will be conducted with 8-10 women and 8-10 men participants in each of the four study arms at each site at enrolment, at 9-12 months postpartum follow-up, and at the end of follow-up. Discussions will focus on the acceptability and feasibility of repeat HTC, involving male partners, the barriers and facilitators to risk reduction behavior, and the determinants of unwanted pregnancies during breastfeeding. We will use theoretic selection for maximum variability in responses. FGDs will also be conducted with 4 midwives and all study counselors at each site, once at baseline and once at the end of follow-up and will focus on the acceptability and feasibility of repeat HTC and of involving male partners.


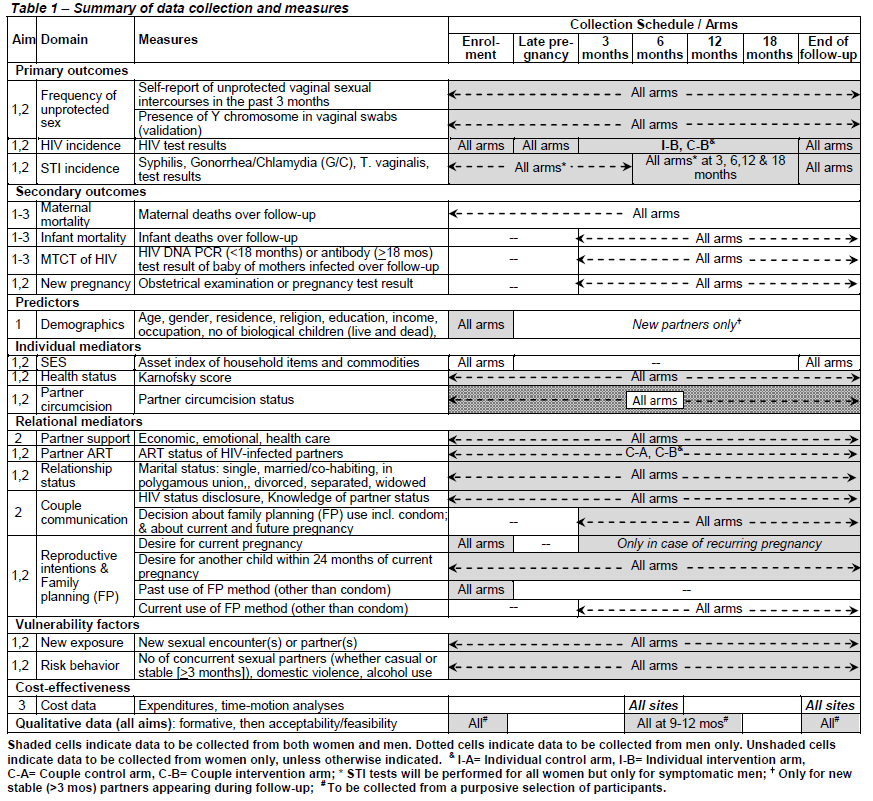


**C.7 Measures**

**C.7.1 Primary outcomes**

The primary outcomes are the frequency of unprotected sex and the incidences of HIV and STI over follow-up. The trial is powered to detect a 10% difference in the frequency of unprotected sex[[2]](#footnote-3) accounting for a 50% loss to follow-up over 2 years. The frequency of unprotected sex will be determined as the self-reported number of episodes of unprotected vaginal intercourse with inconsistent or no condom use in the last 3 months and will be validated by Y chromosome detection in vaginal swabs. Reports by each partner in the couple trial will be treated as two correlated outcomes.

**C.7.2 Secondary outcomes**

Secondary outcomes include the incidence of intended and unintended (recurring) pregnancies, maternal and infant mortality rates, and the rate of MTCT of HIV among infants born to mothers who acquired HIV during follow-up. Post-partum pregnancies will be diagnosed clinically or by urine testing. Infants of HIV-infected mothers will be tested for HIV by DNA PCR before 18 months of age or by rapid HIV antibody test at >18 months of age.

**C.7.3 Predictor variables**

The primary predictor for Aims 1 and 2 will be treatment assignment. Demographic variables including age, gender, residence, religion, education, number of biological children (live and dead), and occupation will be recorded. Individual mediators: SES will be based on an asset index related to the presence or absence of various household items and commodities[^43^](#_ENREF_43) that is used regularly in Uganda Demographic Health Surveys and other research studies; health status will be measured using the Karnofsky performance scale. Relational mediators: Partner emotional support: we will adapt one-dimension of the 19-item Medical Outcomes Study (MOS) Social Support Survey[^44^](#_ENREF_44) that was designed to assess functional social support. This study will only use the emotional/informational support (8 items) dimension of the scale. Partner economic support will be assessed through questions assessing degree of economic dependence. Partner support for health care will be assessed through questions related to material and emotional support specifically related to obstetrical or medical care. Couple communication will be assessed through documentation of HIV serostatus disclosure, knowledge of partner status, and decision-making about current and future pregnancy. Reproductive intentions will be assessed by asking both women and partners about their desire for children within 24 months of the current pregnancy as well as about their past and current use of family planning (FP) methods other than condoms. Unmet need for FP will be calculated as the proportion of women not desiring a child and not using a FP method other than condoms among the total number of women not desiring a child. Vulnerability factors: new partnerships and sexual encounters will be recorded to measure incident exposure. The number of concurrent sexual partners whether casual or stable (> 3 months), history of physical or sexual abuse by the sexual partner or a third party, and alcohol use will be recorded to document high risk behavior.

**C.7.4 Qualitative measurements**

Data collected from focus group discussions and key informant interviews will be triangulated with quantitative data to address: ***(1)*** ***Barriers and facilitators for attending repeat HTC:*** FGDs with women, men and counselors will inform who is and is not attending scheduled HTC and related reasons, as well as the potential limitations of providing retesting and counseling as part of the standard of MCH & PMTCT care services. FGDs will also address potential barriers for service coverage (geographic, cultural, social, financial, educational) and the acceptability and feasibility (from client and counselor perspectives) of retesting women and their partners as well as the potential quality of service women/couples receive. The discussions will address hypothesized structural, relational and individual mediators that may affect re-testing behavior. ***(2) Involving male partners*:** FGDs with women (both individual and in couples), men and health providers will explore issues related to male partners’ roles and involvement in MCH care and HIV prevention. ***(3)*** ***Risk reduction behavior:*** FGDs with women and men will explore what HIV-negative women do to remain negative and the personal, social, economical, cultural and structural facilitators and barriers to different risk reduction options. ***(4)*** ***Unplanned pregnancies at enrolment and during breastfeeding:*** Five women whose pregnancy at enrolment was unintended and five women with an unplanned pregnancy at the end of follow-up in each of the four study arms at each site will be purposively selected and interviewed. Interviews will focus on the circumstances of the unwanted pregnancies in relation to couple communication, risk behavior and HIV prevention.

**C.7.5 Cost data**

Cost data will be collected annually. We will assess costs and cost-efficiency, defined as cost per client served, across all sites and intervention types (individual/couples, WHO repeat HTC/ERHTEC).We will develop a standard cost data collection protocol and manual for gathering expenditure data related to WHO and repeat HTEC in each site. Expenditures will be classified in one of 4 headings: (i) personnel; (ii) recurring goods and services; (iii) capital and equipment; and (iv) building space. In all cases, we will identify costs of providing repeat HTEC and only these costs will be included in the analysis. Capital expenditures such as computers and furniture will be amortized over 5 years of expected useful life, assuming no salvage value. Building space and other in-kind cost will be estimated using the WHO *Cost It* website[^45^](#_ENREF_45), which provides unit prices by region for many resources, including infrastructure, utilities and professionals’ time. .

**C.8 Statistical considerations and analysis plan**

**C.8.1 Preliminary analyses**

We will characterize the sample and compare treatment groups using descriptive statistics and parametric and non-parametric tests as appropriate. We will also assess differences in dropout rates by treatment assignment and sub-trial, as well as baseline and follow-up correlates of dropout, including interactions with treatment assignment.

**C.8.2 Primary analyses**

The primary analysis of the effect of the intervention on the pre-specified primary outcome will be by intent-to-treat without regard to adherence to the intervention, and will be based on pooled data from the individual and couples trials. We will compare counts of unprotected vaginal sex episodes over the last 3 months reported by each participant, including male partners in the couples trial, using log-link negative binomial models. The reporting period for these counts specified by the follow-up questionnaires will be 3 months at all visits and thus can be ignored. Robust “sandwich” standard errors will be used to account for clustering of repeated outcomes within women and couples; over-dispersion of the outcomes will be captured by the quadratic variance-to-mean parameter of the negative binomial model. To reduce potential bias from dropout, follow-up observations will be weighted by the inverse of the estimated probability of remaining in follow-up, given baseline characteristics, treatment assignment, and post-randomization factors; this approach allows us to account for informative post-randomization variables without adjusting away indirect effects of treatment . The model will allow baseline means to vary by site, sub-trial, participant gender, and visit. However, we hypothesize that the multiplicative effect of treatment on the means will be constant. To protect the type-I error rate, the primary analysis will be conducted and reported under this pre-specified hypothesis, modeling the treatment effect by the interaction of treatment assignment with an indicator for follow-up vs baseline. This assumption was used in the sample size calculation, and will be examined in augmented models allowing for heterogeneity and/or trend in the treatment effect across visits. Site, sub-trial (individual vs couples), and participant gender will be modeled as fixed effects. In secondary analyses, we will assess modification of the treatment effect by these factors, yet power will be limited; results will be reported with confidence intervals.

In calculating STI incidence, time-at-risk will be censored for 14 days following initiation of each STI treatment. Treatment effects on incident STIs will be assessed using logistic models, with robust standard errors to account for clustering within women and couples, and inverse weighting to account for informative dropout. HIV incidence will be modeled using pooled logistic models for internal-censored failures, again with robust standard errors and inverse weighting

**C.8.3 Secondary analyses**

In calculating incidence of new pregnancies, time-at-risk will start 60 days following delivery. , Incidence of new pregnancies and MTCT will be modeled as HIV incidence above. For MTCT and new pregnancies, and infant and maternal mortality, the unit of analysis will be newborns and women participants, respectively. **Mediating factors:** The intervention is designed to enhance individual reports of partner economic and emotional support and to enhance couples’ levels of communication, mutual support and decision-making. We expect that as individuals and couple’s levels of psychosocial indices increase, they will be more likely to participate in HIV testing, less likely to engage in HIV sexual risk behavior and less likely to acquire STIs. Specifically, we hypothesize that 1) over time, intervention participants will exhibit higher scores on individual and couple-level psychosocial indices; and 2) psychosocial indices will mediate the relationship between intervention and study outcomes (HIV testing, sexual risk behavior, STI infection). **Individual-level analyses:** We will first assess whether intervention assignment impacts individual-level psychosocial variables using longitudinal mixed models. Second, we will assess whether psychosocial indices impact follow-up retention, sexual risk behavior or STI infection. Third, we will assess whether these changes in psychosocial indices mediate the effect of the intervention on subsequent HIV testing, sexual risk behavior and STI infection using the approach of Kenny[^48^](#_ENREF_48). This approach includes comparing estimates of the effect of the intervention on subsequent HIV testing, sexual behavior and STI infection in models with and without potential mediators. **Dyadic analyses:** In addition to the role of individual-level psychosocial variables on the intervention effect, we will also consider couple-level, (or dyadic) data, looking at actor-partner effects which allow for estimation of the effect of both partners’ psychosocial indices on each other. For example, we will consider the role of a woman’s reported communication on her own outcomes (actor effect) as well as the influence of her male partner’s reported communication on her testing and/or sexual behavior (partner effect). These analyses can be used for both continuous and categorical psychosocial variables. We will examine actor-partner effects for all of the proposed mediating variables on each of the outcomes of interest which will allow us to analyze the role that partners may play in improving the health of each other and their infants.

**C.8.4 Cost and cost-effectiveness**

We will assess the cost of individual and couple-based testing based on the current WHO and our repeat HTC schedule. We will measure the total cost, cost-per-site and cost-per-patient for the intervention and the comparison arms. We will compare the cost of individual and couple-based repeat HTC for both the current WHO recommendation as well as the ERHTEC intervention. We will estimate the cost-effectiveness of repeat HTC by estimating the number of HIV infections averted through these interventions using previously described methods. We will estimate the probability of HIV acquisition for each woman at each time point based on self-reported and validated sexual behavior, HIV and ART status of a woman’s partner and community prevalence of HIV. We will then compare the effectiveness and cost-effectiveness of both individual and couple-based repeat HTC to the controls.

**C.8.5 Sample size and power**

We will enroll 410 individual women and 410 couples. The combined sample of 1,230 participants will provide 80% power with a type-I error rate of 5% (2-sided) to detect a 10% reduction in the number of unprotected vaginal sex episodes in the repeat HTC arms, net of post-randomization reductions in the control arms. The sample size calculation accounts for within-person and within-couple correlation, estimated at 0.3; a total of 7 assessments, including baseline; 50% cumulative loss to follow-up over 18-months; a control-arm reduction in frequency of 30%; baseline frequency of 5 unprotected episodes per 3 months; and over-dispersion of 2 with respect to a Poisson distribution, used as an approximation to the negative binomial model. To obtain sample size, we modeled the covariance matrix of the parameter estimates in the model used for the primary analysis as the inverse of the sum over participants of matrices X’WX, where X is a matrix of predictor variables specifying time, treatment, and their interaction, with a row for each visit, and W is the inverse of the covariance matrix of the repeated over-dispersed Poisson outcomes, approximated as compound symmetric; for couples, X has two rows per visit. Dropout was accounted for by varying the number of rows of X across participants. This gave an estimate of the SE of the treatment effect estimate, as required in the standard formula for power with a normally-distributed test statistic.

Assumptions above regarding participant retention rate (50%), within-couple correlation (0.3), baseline frequency of unprotected episodes (5 per 3 months), the average reduction in episodes in both arms taken together, and over-dispersion (2), all of which can be estimated without using current information on the treatment effect, will be checked midway through enrolment, i.e. once 50% of the enrolment target has been reached. Power under the planned sample size will then be reviewed using updated estimates for these parameters, under the original assumptions about the treatment effect. If power appears to be low or too high, increasing or decreasing the sample size will be discussed with the study investigators. Because this assessment is made without using current information on the treatment effect, increases or decreases in the sample size can be made with no inflation of the type-I error rate^53.54^.

In case of persistent difficulties in enrolling couples, we will consider enrolling more single women. Given our assumptions about within-couple correlation, we would only have to enroll 1.7 extra single women for each missing couple. This would weaken inferences about effects in couples, thus would only be considered as a last resort, should couple enrolment fall short of the initial target.

**C.8.6 Qualitative data analysis**

Focus group discussions and in-depth interviews will be transcribed, translated into English, and coded. Analysis will include 3 distinct stages: reducing the data into meaningful segments, naming the segments, combining them into categories or themes and displaying and making comparisons[^55^](#_ENREF_53) to be used for thematic coding as the primary analytic strategy. After reading 4 transcripts, the analysis team will collaboratively develop a codebook of themes based on interview topics as well as those emerging from the data. Two more transcripts will then be reviewed to include additional topic areas and themes. This process will be repeated until the code-book reaches a stage where no new themes or topic areas emerge. To ensure inter-rater consistency, the analysis team will compare their individual coding of the same transcripts and calculate a coding concordance. All transcripts will then be coded using the final version of the codebook and merged using NVivo software (version 10.0, QSR International, Victoria, Australia). Using NVivo, themes will be summarized across participants. Analysis will focus on identifying dominant explanations for themes identified. Interactive discussions will be held to validate data interpretations and resolve any discrepancies. Our analysis will highlight both, issues described in the study design as well as issues emerging from participant engagement. Trustworthiness will be ensured through the use of different methods for data collection and triangulation of data. A monthly debriefing of the counselors will be held to review counseling notes, triangulating these with relevant quantitative measures

**C.8.7 Data quality and management**

SOPs will be developed for all data management process and procedures to ensure consistency and quality of data. **Quantitative data** will be collected and entered by research staff on CRFs and/or Android electronic tablets (hand-held computers) using “ODK Collect”, a computer-assisted personal interviewing (CAPI) software. The data will be uploaded via internet onto a secure central server according to SOPs. The central web-based server is located in Munich, Germany (http://www.giga-international.com) and is protected by multiple security layers. The data will be stored securely in the central server and backed up into secure central MySQL-server databases at MU-JHU Care in Kampala and at UCSF in San Francisco. These servers are located in access-limited rooms within MU-JHU and UCSF facilities and each computer and each database used to store the data will be protected by separate passwords. Procedures to promote data quality will include skip patterns and range and logical checks built into the ODK Collect entry program, and data validation and cleaning at the point of collection using two levels of quality controls (same-day check and feed-back by data collector and Study Coordinator) according to SOPs.

**Qualitative data** will be collected manually and on a voice recorder and will be transcribed into laptop computers by data clerks and/or research assistants for subsequent coding in NVivo software and analysis. All discussions will be conducted in the local languages, will last no more than 90 minutes, and will be digitally recorded and transcribed into English. Interviewers/research assistants will have experience in qualitative methods including in-depth interviewing and probing where appropriate and relevant. Digital recordings will be transferred to secure computers and deleted from source computers.

All data clerks and managers will have received training on the requirements of strict confidentiality regarding patient identifying information, and laptop security. To protect detailed health and personal information data, study record keeping and access to participant identifying information will follow strict written SOPs and data will be subjected to a variety of quality control procedures. All records will be kept on password protected computers at MU-JHU and UCSF. Participants will be identified primarily by their study number. All CRFs and identifiers will be kept in individual files in secured, access-limited rooms at the sites. Participant names and addresses will be stripped from the database prior to analysis. No individual identifier will be used in any reports or publications resulting from the study. Study investigators and coordinators will continuously mentor and monitor staff to ensure these procedures are followed according to SOPs in order to ensure data quality and confidentiality.

# D. HUMAN SUBJECTS PROTECTION

## D.1 Ethical and regulatory considerations

The study will be conducted in compliance with country specific laws and regulatory requirements. The conduct of the study will also adhere to the GCP principles laid out by the International Conference on Harmonization (ICH) and the Declaration of Helsinki

The protocol and informed consent form will be reviewed and approved by the local IRB in Uganda, UNCST and at UCSF prior to enrollment or carrying out any study procedures. The PI will keep the IRB informed of the progress of the study on a regular basis including any unexpected untoward events, at minimum once a year according to the IRB requirements.

Written informed consent will be obtained from the study participants prior to any protocol-specified procedures being conducted.

To maintain confidentiality, unique study participant identification numbers will be used to identify the subject’s laboratory specimens, source documents, CRF, study reports, etc. All study records will be maintained in a secured location and consents and other identifying information will be kept in a locked cabinet in a room with restricted access which is locked during non business hours.

## D.2 Institutional Review Board (IRB) or Independent Ethics Committee (IEC)

All the documents the IRB/IEC may need to fulfill its responsibilities, such as the protocol, protocol amendments, information concerning subject recruitment, payment or compensation procedures, etc., will be submitted to the IRB/IEC by the investigator. The IRB’s/IEC’s written, unconditional approval of the study protocol and the informed consent form will be in the possession of the investigator/clinical site staff prior to the conduct of any protocol-specified procedures.

Modifications to the protocol will not be implemented without prior written IRB/IEC approval except when necessary to eliminate immediate hazards to the subjects or when the modification involves only logistical or administrative aspects of the study. Such logistical or administrative modifications will be submitted to the IRB/IEC in writing by the investigator, and a copy of the correspondence to verify the submission will be maintained.

The investigator must inform the IRB/IEC of modifications to the informed consent form or any other documents previously submitted for review/approval, of any new information that may adversely affect the safety of the subjects or the conduct of the study, provide an annual update and/or request for re-approval, and advise the IRB/IEC when the study has been completed.

## D.3 Informed Consent

The principles of informed consent will follow ICH-GCP. Informed consent will be documented in writing on a consent form approved by the IRB/IEC in English and translated into the local language.

All relevant information should be provided in both oral and written form in a way that is understandable to the legal guardian of the subject. Ample time and opportunity must be given for the subject to inquire about details of the study. The written consent document will embody the elements of informed consent as described in the Declaration of Helsinki and will also comply with local regulations.

The study counselor will explain the nature of the study, answer study questions and obtain informed consent from the study participants. The original, signed informed consent form for each subject will be maintained by the investigator as part of the subject’s study records. A copy of the signed informed consent form will be offered to each the study participants.

## D.4 Study Completion

The investigator will notify the IRB/IEC when the study has been completed and will provide copies of conference abstracts and publications resulting from the study.

## D.5 Publications and dissemination of study findings

Data analyses and publications will be under the direction of the PI. After briefing the MOH, primary study outcomes will be submitted to the local (SOMREC) and US (UCSF CHR) IRBs for clearance. Once cleared, findings will be presented at a special dissemination workshop involving Kitgum and Kampala stakeholders including the hospitals’ staff, district and MOH leadership, as well as community representatives and non-governmental and community-based organizations. Results will also be summarized in a leaflet written in lay language and translated into Luganda and Luo for distribution to study participants after ethical clearance by SOMREC and UCSF CHR is obtained. Lastly, manuscripts and abstracts will be prepared for submission to national and international scientific meetings as well as peer-reviewed journals.

# E. TIMELINE

|  | **Year 1**  **(2011-12)** | | | **Year 2**  **(2012-13)** | | | | **Year 3**  **(2013-14)** | | | | | **Year 4**  **(2014-15)** | | | | **Year 5**  **(2015-16)** | | | |
| --- | --- | --- | --- | --- | --- | --- | --- | --- | --- | --- | --- | --- | --- | --- | --- | --- | --- | --- | --- | --- |
| Activities Quarter | Q4 | Q1 | Q2 | Q3 | Q4 | Q1 | Q2 | | Q3 | Q4 | Q1 | Q2 | Q3 | Q4 | Q1 | Q2 | Q3 | Q4 | Q1 | Q2 |
| IRB, MOU, planning & CRF design |  |  |  |  |  |  |  | |  |  |  |  |  |  |  |  |  |  |  |  |
| Staff recruitment & site preparations |  |  |  |  |  |  |  | |  |  |  |  |  |  |  |  |  |  |  |  |
| Staff and peer training |  |  |  |  |  |  |  | |  |  |  |  |  |  |  |  |  |  |  |  |
| MULAGO: Screening & enrolment |  |  |  |  |  |  |  | |  |  |  |  |  |  |  |  |  |  |  |  |
| MULAGO: Follow-up |  |  |  |  |  |  |  | |  |  |  |  |  |  |  |  |  |  |  |  |
| KITGUM: Screening & enrolment |  |  |  |  |  |  |  | |  |  |  |  |  |  |  |  |  |  |  |  |
| KITGUM: Follow-up |  |  |  |  |  |  |  | |  |  |  |  |  |  |  |  |  |  |  |  |
| Data cleaning and analysis |  |  |  |  |  |  |  | |  |  |  |  |  |  |  |  |  |  |  |  |

# References

# Appendix A - Formative focus group discussion points for women (n=8-10 participants per group)

**Outline**

Group 1 - HIV-negative women presenting in ANC clinics individually – Mulago

Group 2 - HIV-negative women presenting with their partner - Mulago

Group 3 - women’s male partners – Mulago

Group 4 - HIV-negative women presenting in ANC clinics individually – Kitgum

Group 5 - HIV-negative women presenting with their partner - Kitgum

Group 6 - women’s male partners – Kitgum

Discussion focus: Primary HIV prevention for pregnant and lactating women

**Awareness of the risks**: What has the group ever heard with regards to:

- the concept of primary HIV prevention for pregnant and lactating women?
- the four pillars/prongs of prevention of mother-to-child transmission of HIV (PMTCT)?

**Understanding of the risks:**

- what puts a pregnant or lactating women at risk for HIV?
- Probe on potential increased risks for a pregnant or breastfeeding woman to become HIV-infected? Why/why not? Which are the increased risks?

What are the risks involved with:

- being an HIV-infected pregnant woman?
- being an HIV-infected breastfeeding mother?
- becoming HIV-infected while pregnant?
- becoming HIV-infected while breastfeeding?

**Beliefs:**

- What causes some women who have unprotected sex to become HIV-infected and others who also have unprotected sex to remain uninfected?
- What causes a pregnant or breastfeeding woman to become HIV-infected?
- What is the role of the men (spouses/fathers) in protecting their wives from becoming infected?
- What is the role of the men (spouses/fathers) in making their wives become infected?

**Attitudes**:

- How do doctors / midwives / traditional birth attendants help/not help pregnant or breastfeeding women protect themselves from HIV?
- How easy is it to use condoms in your couple / community? What are some barriers and facilitators to condom use? Any experiences with female condoms?

**Practices:**

- What are the cultural practices around sexual activity during pregnancy and breastfeeding? (probe about whether men leave the house / abstain / have other sexual partners)
- What are your experiences in being exposed to HIV while pregnant or breastfeeding?
- What are your experiences in protecting yourself and your baby from becoming HIV-infected while pregnant or breastfeeding?
- Please describe how easy is it to get condoms in your community?
- How many have used condoms consistently during pregnancy or breastfeeding? Please describe barriers and facilitators

**Challenges and opportunities**:

- What can a pregnant or breastfeeding woman do to avoid becoming HIV infected?
- What can a man do to help protect his pregnant or breastfeeding wife to become HIV infected?
- What services do pregnant or breastfeeding women receive to avoid becoming HIV infected? What services are missing/needed?

# Appendix B - Counseling Guide Outline

The guide will be adapted from the national HIV counseling guide and the CDC couple counseling guide and will focus on the concept of keeping the baby and family safe from HIV throughout pregnancy and breastfeeding, emphasizing the critical steps to achieve this goal, including the need to:

- check the family’s HIV status regularly
- adopt sustained preventive behaviors
- feed the baby safely and adequately
- and plan for future pregnancies

It will clarify the concepts of acute and incident infection , explain the “window” period, discuss serodiscordance and its implications and all available ways to minimize transmission risks, and will integrate the standard safe motherhood and safe infant feeding messages aimed at all pregnant and breastfeeding women.

**The guide will address six possible retesting scenarios for individual women or couples**:

1) individual woman remaining HIV-negative

2) individual woman with incident HIV infection

3) sero-concordant HIV-negative couple

4) sero-discordant couple with HIV-infected woman

5) sero-discordant couple with HIV-infected man; and

6) sero-concordant HIV-positive couple.

For each scenario, the guide will emphasize:

- primary prevention for the individual(s) remaining HIV-negative
- positive prevention for the HIV-positive individual(s)
- disclosure, communication, sex behavior, PMTCT prophylaxis and HIV care issues.

For primary prevention, the guide will address risk behaviors and sources of potential exposure to HIV since the last test, specific vulnerabilities and risks associated with different stages of the pregnancy, delivery and postpartum periods including cultural practices and beliefs associated with sexual activity during pregnancy and breastfeeding and resumption of sexual activity after delivery.

The guide will emphasize “positive living” for both positive and primary prevention (protected sex, adequate nutrition, psycho-social support, male circumcision, avoidance of unwanted pregnancies).

In the case of serodiscordance, the guide will emphasize ART for the HIV-infected couple member, and all methods recommended for pre-exposure prophylaxis at the time of the trial such as tenofovir-based pre-exposure prophylaxis and/or microbicide gel for the HIV-uninfected couple member if recommended.

A section specific to couples will incorporate this information with the guidelines currently in use in Uganda for couple HTC including establishing the conditions for couple counselling (consent, open discussion, receiving test results together, respecting the confidentiality of shared results, joint decision about disclosure to third party), the expectations from, and roles and responsibilities of each partner (respect, equal participation, responsiveness, openness, mutual support), and the particular issues related to sero-concordance and serodiscordance of results (keeping the HIV-negative partner uninfected, ongoing care and support of the HIV-positive partner, and assessing the risk of violence or abuse and strategies for avoiding it).

For couples becoming newly serodiscordant, counselors will focus on addressing the concepts, myths and misperceptions around serodiscordance and will discuss coping strategies including couple communication and mutual support. For both serodiscordant and HIV-positive seroconcordant couples, counselors will further emphasize risk reduction, PMTCT, and address reproductive health issues, including family planning for infected women.

# Appendix C - Schedule of events - WOMEN

ERHTEC = Extended Repeat HIV

Testing and Enhanced Counseling

HTC = HIV testing & counseling

STI = Sexually Transmitted Infections

**CONTROLS**

**INTERVENTION**

**- Blood draw + vaginal swab**

**- Baseline questionnaire**

**- STI TC**

**- Blood draw + vaginal swab**

**- Baseline questionnaire**

**- STI TC + Enhanced counseling**

**BASELINE**

**- Blood draw + vaginal swab**

**- Follow-up questionnaire-**

**- Repeat standard HTC**

**- Blood draw + vaginal swab**

**- Follow-up questionnaire**

**- ERHTEC HIV & STI TC**

**POST-NATAL VISITS**

**- ERHTEC (counseling only):**

**Infant feeding**

**Risk reduction**

**Family planning**

**- Blood draw + vaginal swab**

**- Follow-up questionnaire-**

**- Repeat standard HTC**

**- Blood draw + vaginal swab**

**- Follow-up questionnaire**

**- ERHTEC: HIV & STI TC**

**- Blood draw + vaginal swab**

**- Follow-up questionnaire**

**- ERHTEC: HIV & STI TC**

**- Blood draw + vaginal swab**

**- Follow-up questionnaire-**

**- Repeat standard HTC**

**- ERHTEC (counseling only):**

**Infant feeding**

**Risk reduction**

**Family planning**

**- Blood draw + vaginal swab**

**- Follow-up questionnaire**

**- ERHTEC: HIV & STI TC**

**- Blood draw + vaginal swab**

**- Follow-up questionnaire**

**- Repeat standard HTC**

**- ERHTEC (counseling only):**

**Infant feeding**

**Risk reduction**

**Family planning**

**- Blood draw + vaginal swab**

**- Follow-up questionnaire**

**- ERHTEC: HIV & STI TC**

**- Follow-up questionnaire**

**- Blood draw + vaginal swab**

**- Repeat standard HTC**

**- ERHTEC (counseling only):**

**Infant feeding**

**Risk reduction**

**Family planning counseling**

**Final Assessment (24 mos or end of BF)**

**- Blood draw + vaginal swab**

**- Final questionnaire**

**- ERHTEC: HIV & STI TC**

**- Blood draw + vaginal swab**

**- Final questionnaire**

**- ERHTEC: HIV + STI TC**

**^✝^** The timing and events of the late pregnancy and 6-week visits will depend of the gestational age of the woman at enrolment (see Protocol C.5.1)

***Appendix C (continued)* – Schedule of events - MEN**

ERHTEC = Extended Repeat HIV

Testing and Enhanced Counseling

HTC = HIV testing & counseling

STI = Sexually Transmitted Infections

**CONTROLS**

**INTERVENTION**

**- Blood draw + urethral swab**

**- Baseline questionnaire**

**- STI TC**

**- Blood draw + urethral swab**

**- Baseline questionnaire**

**- STI TC + Enhanced counseling**

**BASELINE**

**- Blood draw + urethral swab***

**- Follow-up questionnaire**

**- Repeat standard HTC**

**- Blood draw + urethral swab**

**- Follow-up questionnaire**

**- ERHTEC: HIV & STI TC**

**POST-NATAL VISITS**

**- ERHTEC (counseling only):**

**Infant feeding**

**Risk reduction**

**Family planning**

**- Blood draw + urethral swab***

**- Follow-up questionnaire**

**- Repeat standard HTC**

**- Blood draw + urethral swab**

**- Follow-up questionnaire**

**- ERHTEC: HIV & STI TC**

**- Blood draw + urethral swab***

**- Follow-up questionnaire**

**- Repeat standard HTC**

**- Blood draw + urethral swab**

**- Follow-up questionnaire**

**- ERHTEC: HIV & STI TC**

**- ERHTEC (counseling only):**

**Infant feeding**

**Risk reduction**

**Family planning counseling**

**- Blood draw + urethral swab**

**- Follow-up questionnaire**

**- ERHTEC: HIV & STI TC**

**- Blood draw + urethral swab***

**- Follow-up questionnaire**

**- Repeat standard HTC**

**- ERHTEC (counseling only):**

**Infant feeding**

**Risk reduction**

**Family planning counseling**

**- Blood draw + urethral swab**

**- Follow-up questionnaire**

**- ERHTEC: HIV & STI TC**

**- Blood draw + urethral swab***

**- Follow-up questionnaire**

**- Repeat standard HTC**

**- ERHTEC (counseling only):**

**Infant feeding**

**Risk reduction**

**Family planning counseling**

**cotra**

**Final Assessment (24 mos or end of BF)**

**- Blood draw + urethral swab**

**- Final questionnaire**

**- ERHTEC: HIV & STI TC**

**- Blood draw + urethral swab**

**- Final questionnaire**

**- ERHTEC: HIV + STI TC**

**^✝^** The timing and events of the late pregnancy and 6-week visits will depend of the gestational age of the woman at enrolment (see Protocol C.5.1)

* Urine or swab for STI testing to be collected only from symptomatic men at these visits

***Appendix C (cont’d*) - Summary of events and measurements by study arms and visits**

|  | **Controls** | | **All** | | | **Intervention arm (ERHTEC)** | | |
| --- | --- | --- | --- | --- | --- | --- | --- | --- |
| **Follow-up** | **Standard counseling** | **Standard HIV testing & counseling** | **STI testing** | **Behavioral questionnaire, blood draws + vaginal/urethral swabs** | **Qualitative intervention assessment** | **HIV testing & enhanced counseling** | | **Risk reduction counseling,**  **Infant feeding/nutrition education & Family planning counseling** |
| Formative |  |  |  |  | **X** |  | |  |
| Baseline | **X** | **X** | **X** | **X** |  | **X** | | **X** |
| Late pregnancy | **X** | **X** | **X*** | **X** |  | **X** | | **X** |
| *6 weeks✝* | ***x*** |  |  |  |  |  | | ***X*** |
| 3 months | **X** | **X** | **X*** | **X** |  | **X** | | **X** |
| 6 months | **X** | **X** | **X*** | **X** |  | **X** | | **X** |
| *9 months✝* |  |  |  |  |  |  | | ***X*** |
| 12 months | **X** | **X** | **X*** | **X** | **X** | **X** | | **X** |
| *15 months✝* |  |  |  |  |  |  | | ***X*** |
| 18 months | **X** | **X** | **X*** | **X** |  | **X** | | **X** |
| *21 months✝* |  |  |  |  |  |  | | ***X*** |
| 24 months**^+^** | **X** | **X** | **X** | **X** | **X** | **X** | | **X** |
|  |  | **Measurements** | | | | |  | |

✝ **Retention visits:
-** Participants in the intervention arms will receive enhanced counseling but blood and vaginal swabs or urine samples will not be
 collected unless deemed necessary by the clinician.
- Participants in the control arms will receive standard counseling when study visits correspond to scheduled post-natal visits but
 will not be asked to come back otherwise until their final follow-up visit.
- No questionnaire will be administered during retention visits.

**^#^ Only symptomatic men will be tested for STIs at these time points**

**^+^ 24 months or** **6 weeks after the end of breastfeeding**, **whichever occurs first**

# Appendix D: End of study qualitative intervention assessments

**Theme: Acceptability and feasibility of the ERHTEC intervention**

**a) Focus group discussion points – Outline / Staff**

**Acceptability:**

How did women and men participants receive the intervention?

What needs did it correspond to?

What additional needs/interests did it generate?

**Feasibility:**

How was the intervention received by the providers who implemented it?

How practical was the intervention for the participants?

What were the constraints (time, language, men involvement, tracing defaulters)?

What changes did the intervention imply in the organization of the clinic/service?

How could the intervention become part of routine MNCH/PMTCT care?

**Challenges:**

What were the main challenges to the participants’ acceptability of the intervention?

What were the main challenges to the staff’s acceptability of the intervention?

**Opportunities:**

What could be done to improve the content of the ERHTEC intervention?

What could be done to increase the acceptability and feasibility of the intervention?

**Scalability:**

What is needed for this intervention to become part of routine MNCH/PMTCT care at all health center levels?

What would it take to integrate the intervention into routine MNCH/PMTCT care?

**b) Key informant interview guide – Outline / participants**

**Acceptability:**

How was the intervention received by participant?

What needs did it correspond to?

What additional needs/interests did it generate?

What were the motivators that helped participants remain engaged in the intervention?

(For couples) How easy/difficult was it to keep the partner engaged?

**Feasibility:**

How convenient/inconvenient was the intervention to participant?

How easy/difficult was it to adhere to the visit schedule?

What were the constraints (time, language, men involvement, missing visits)?

What changes did the intervention cause in the participants’ life? Types, scope and quality of changes

**Challenges:**

What were the main challenges to participants’ acceptability of the intervention?

What were the main challenges to participants’ adherence to the intervention?

**Opportunities:**

What could be done to improve the content of the ERHTEC intervention?

What could be done to increase the acceptability and feasibility of the intervention?

What other intervention could help participants to implement primary HIV prevention?

**Scalability:**

What services do participants expect to receive as part of routine MNCH/PMTCT care after having experienced the ERHTEC intervention?

1. This was done in response to a comment raised by a NIH reviewer who stated: *“[…] it is not clear how feasible it is to follow up couples for two or more years. […] The question is attrition rate over time – the power analysis assumes 30%, but a sensitivity analysis up to 50% would be a good idea“.* In response to this comment, we increased our attrition assumption in our sample size calculation from 30% (original submission) to 50% to ensure sufficient power for the primary endpoint comparisons.: [↑](#footnote-ref-2)
2. The study could not be powered on HIV incidence because the sample sizes required to detect significant decreases in HIV or STI incidences would be too large given the baseline incidence estimates in the population to be studied. The current estimated annual incidence of HIV in pregnant women in Uganda is <2% while syphilis, Gonorrhea/Chlamydia and Trichomonas vaginalis prevalence rates are <4% among HIV-negative women, resulting in sample sizes >2,500 for a 30% reduction over 2 years, which is beyond the scope of this study. We also considered a composite STI primary endpoint but the sample size remained >1,500. [↑](#footnote-ref-3)
