# Supplementary material for: Primary HIV prevention in pregnant and lactating Ugandan women: A randomized trial
Source: PLoS One. 2019 Feb 25;14(2):e0212119. doi: 10.1371/journal.pone.0212119 (PMC6388930; doi:10.1371/journal.pone.0212119)
Supplement: S2 Table — (PDF) [file pone.0212119.s004.pdf]

ANALYSES RESTRICTED TO WOMEN REPORTING NO CONDOM USE AT BASELINE

S2.1 nvi1 (frequency of vaginal sex, last 3 months), by visit: N, median, IQR

treatment assignment	S&E	L&D/6wks	3 mos	6 mos	12 mos	18 mos	24 mos
intervention	342	218	197	220	231	203	231
	12.0	9.0	6.0	10.0	16.0	20.0	16.0
	6.0	4.0	3.0	5.0	6.0	6.0	6.0
	24.0	18.0	15.0	24.0	32.0	36.0	35.0
control	338	207	190	234	240	209	241
	13.0	7.0	6.0	10.0	15.0	24.0	20.0
	7.0	3.0	3.0	5.0	7.0	8.0	7.0
	24.0	16.0	15.0	25.0	30.0	36.0	36.0

using robust SEs to get p-values
difference in average change by treatment assignment: P=0.87
restricted to months 3-24: P=0.60
difference in average follow-up level by treatment assignment: P=0.61
restricted to months 3-24: P=0.33
difference in linear trend by treatment assignment: P=0.10
restricted to months 3-24: P=0.19

S2.2 nviuc1: condom use for vaginal sex, last 3 months, by visit

txr = intervention

condom use for vaginal sex, last 3 months, by visit	S&E	L&D/6wks	3 mos	6 mos	12 mos	18 mos	24 mos	Total
always	0	8	21	23	20	12	17	101
	0.00	3.72	10.82	10.45	8.77	5.97	7.42	6.20
sometimes	0	17	21	39	50	45	33	205
	0.00	7.91	10.82	17.73	21.93	22.39	14.41	12.58
never	342	190	152	158	158	144	179	1,323
	100.00	88.37	78.35	71.82	69.30	71.64	78.17	81.22
Total	342	215	194	220	228	201	229	1,629
	100.00	100.00	100.00	100.00	100.00	100.00	100.00	100.00

txr = control

condom use for vaginal sex, last 3 months, by visit	S&E	L&D/6wks	3 mos	6 mos	12 mos	18 mos	24 mos	Total
always	0	4	23	24	18	13	8	90
	0.00	1.96	12.23	10.26	7.53	6.40	3.39	5.48
sometimes	0	12	23	24	29	34	34	156
	0.00	5.88	12.23	10.26	12.13	16.75	14.41	9.50
never	338	188	142	186	192	156	194	1,396
	100.00	92.16	75.53	79.49	80.33	76.85	82.20	85.02
Total	338	204	188	234	239	203	236	1,642
	100.00	100.00	100.00	100.00	100.00	100.00	100.00	100.00

difference in average change by treatment assignment: P=0.51
restricted to months 3-24: P=0.72
difference in average follow-up level by treatment assignment: P=0.047
restricted to months 3-24: P=0.07
difference in linear trend by treatment assignment: P=0.84
restricted to months 3-24: P=0.76

S2.3 pviwc (proportion of vaginal sex episodes with condoms), by visit: N, mean, SD

treatment assignment	visit S&E	L&D/6wks	3 mos	6 mos	12 mos	18 mos	24 mos
intervention	342	215	194	220	228	201	229
	0.0	7.1	15.6	17.6	17.5	14.9	13.2
	0.0	22.2	33.3	33.8	32.5	29.4	30.1
control	338	204	188	234	239	203	236
	0.0	4.4	17.6	14.8	12.6	11.9	10.3
	0.0	17.2	35.0	32.7	29.9	27.8	25.4

difference in average change by treatment assignment: P=0.14
restricted to months 3-24: P=0.21
difference in average follow-up level by treatment assignment: P=0.15
restricted to months 3-24: P=0.21
difference in linear trend by treatment assignment: P=0.67
restricted to months 3-24: P=0.82

S2.4 pvinc (proportion of vaginal sex episodes w/o condoms), by visit: N, mean, SD

treatment assignment	visit S&E	L&D/6wks	3 mos	6 mos	12 mos	18 mos	24 mos
intervention	342	215	194	220	228	201	229
	100.0	92.9	84.4	82.4	82.5	85.1	86.8
	0.0	22.2	33.3	33.8	32.5	29.4	30.1
control	338	204	188	234	239	203	236
	100.0	95.6	82.4	85.2	87.4	88.1	89.7
	0.0	17.2	35.0	32.7	29.9	27.8	25.4

difference in average change by treatment assignment: P=0.14
restricted to months 3-24: P=0.21
difference in average follow-up level by treatment assignment: P=0.15
restricted to months 3-24: P=0.21
difference in linear trend by treatment assignment: P=0.67
restricted to months 3-24: P=0.82

S2.5 proportion of vaginal sex episodes with condoms, by visit: N, mean, SD

treatment assignment	visit S&E	L&D/6wks	3 mos	6 mos	12 mos	18 mos	24 mos
intervention	342	215	194	220	228	201	229
	0.0	7.1	15.6	17.6	17.5	14.9	13.2
	0.0	22.2	33.3	33.8	32.5	29.4	30.1
control	338	204	188	234	239	203	236
	0.0	4.4	17.6	14.8	12.6	11.9	10.3
	0.0	17.2	35.0	32.7	29.9	27.8	25.4

inferences using binomial model
difference in average change by treatment assignment: P=0.84
convergence not achieved
restricted to months 3-24: P=0.
difference in average follow-up level by treatment assignment: P=0.09
restricted to months 3-24: P=0.14
difference in linear trend by treatment assignment: P=0.27
restricted to months 3-24: P=0.76

S2.6 nvincl(frequency of vaginal sex w/o condoms, last 3 months), by visit: N, median, IQR

treatment assignment	visit S&E	L&D/6wks	3 mos	6 mos	12 mos	18 mos	24 mos
intervention	342	215	194	220	228	201	229
	12.0	8.0	5.0	9.0	12.0	15.0	14.0
	6.0	3.0	2.0	3.0	4.0	4.0	4.0
	24.0	18.0	14.0	20.0	30.0	34.0	32.0
control	338	204	188	234	239	203	236
	13.0	7.0	5.0	10.0	12.0	20.0	19.0
	7.0	3.0	2.0	3.0	5.0	5.0	6.0
	24.0	15.5	13.5	24.0	25.0	36.0	35.5

using robust SEs to get p-values
difference in average change by treatment assignment: P=0.50
restricted to months 3-24: P=0.32
difference in average follow-up level by treatment assignment: P=0.21
restricted to months 3-24: P=0.10
difference in linear trend by treatment assignment: P=0.06
restricted to months 3-24: P=0.17

omitting women in couples

S2.7 nvincl(frequency of vaginal sex w/o condoms, last 3 months), by visit: N, median

treatment assignment	visit S&E	L&D/6wks	3 mos	6 mos	12 mos	18 mos	24 mos
intervention	151	92	73	82	92	81	94
	12.0	6.0	5.0	7.0	17.0	20.0	16.0
control	151	80	78	97	106	88	102
	12.0	7.0	4.0	10.0	14.0	20.0	21.0

difference in average change by treatment assignment: P=0.58
restricted to months 3-24: P=0.46
difference in average follow-up level by treatment assignment: P=0.67
restricted to months 3-24: P=0.91
difference in linear trend by treatment assignment: P=0.65
restricted to months 3-24: P=0.26

S2.8 lviucl(condom use at last vaginal sex, last 3 months), by visit: N, n, proportion

treatment assignment	visit S&E	L&D/6wks	3 mos	6 mos	12 mos	18 mos	24 mos
intervention	342	218	197	219	231	203	231
	0.000	19.000	25.000	35.000	34.000	28.000	29.000
	0.000	0.087	0.127	0.160	0.147	0.138	0.126
control	338	207	190	234	240	209	241
	0.000	11.000	27.000	30.000	24.000	21.000	13.000
	0.000	0.053	0.142	0.128	0.100	0.100	0.054

difference in average follow-up level by treatment assignment: P=0.04
restricted to months 3-24: P=0.06
difference in linear trend by treatment assignment: P=0.31
restricted to months 3-24: P=0.10
