# Supplementary material for: Primary HIV prevention in pregnant and lactating Ugandan women: A randomized trial
Source: PLoS One. 2019 Feb 25;14(2):e0212119. doi: 10.1371/journal.pone.0212119 (PMC6388930; doi:10.1371/journal.pone.0212119)
Supplement: S3 Table — (PDF) [file pone.0212119.s005.pdf]

S3_Tables – Any STI detected over follow-up, by site, enrolment status and visit (N, n, proportion)

S3.1: BY SITE

Treatment, stratified by site	S&E	L&D/6wks	3 mos	6 mos	12 mos	18 mos	24 mos
KA,Control	205	172	149	146	136	115	137
	12	8	4	12	15	7	18
	.0585	.0465	.0268	.0822	.11	.0609	.131
KA,ERHTEC	204	201	153	145	136	111	143
	9	10	13	8	10	15	17
	.0441	.0498	.085	.0552	.0735	.135	.119
KI,Control	204	202	171	172	177	166	179
	4	10	5	9	13	18	35
	.0196	.0495	.0292	.0523	.0734	.108	.196
KI,ERHTEC	206	222	172	175	182	170	171
	6	8	10	7	15	22	27
	.0291	.036	.0581	.04	.0824	.129	.158

(Column group heading: BY SITE — visit)

KA: Kampala / KI: Kitgum
Tests for modification of treatment effects by site, using logistic model
effect on average change: P=0.2497
effect on average follow-up level: P=0.6888
effect on linear trend: P=0.3072

S3.1: BY ENROLMENT STATUS

any_sti (any STI detected), by visit: N, n, proportion

Treatment, stratified by couple	S&E	L&D/6wks	3 mos	6 mos	12 mos	18 mos	24 mos
INDIV,Control	205	179	153	149	150	133	154
	7	6	2	11	12	11	28
	.0341	.0335	.0131	.0738	.08	.0827	.182
INDIV,ERHTEC	204	214	155	148	151	134	146
	10	10	15	9	15	18	25
	.049	.0467	.0968	.0608	.0993	.134	.171
CPLE,Control	204	195	167	169	163	148	162
	9	12	7	10	16	14	25
	.0441	.0615	.0419	.0592	.0982	.0946	.154
CPLE,ERHTEC	206	209	170	172	167	147	168
	5	8	8	6	10	19	19
	.0243	.0383	.0471	.0349	.0599	.129	.113

(Column group heading: BY ENROLMT STATUS — visit)

INDIV: Women enrolled individually
CPLE: Women enrolled with their partner
Tests for modification of treatment effects by couple, using logistic model
effect on average change: P=0.4867
effect on average follow-up level: P=0.1404
effect on linear trend: P=0.5335
