# Supplementary figures and images for: Primary HIV prevention in pregnant and lactating Ugandan women: A randomized trial
Source: PLoS One. 2019 Feb 25;14(2):e0212119. doi: 10.1371/journal.pone.0212119 (PMC6388930; doi:10.1371/journal.pone.0212119)

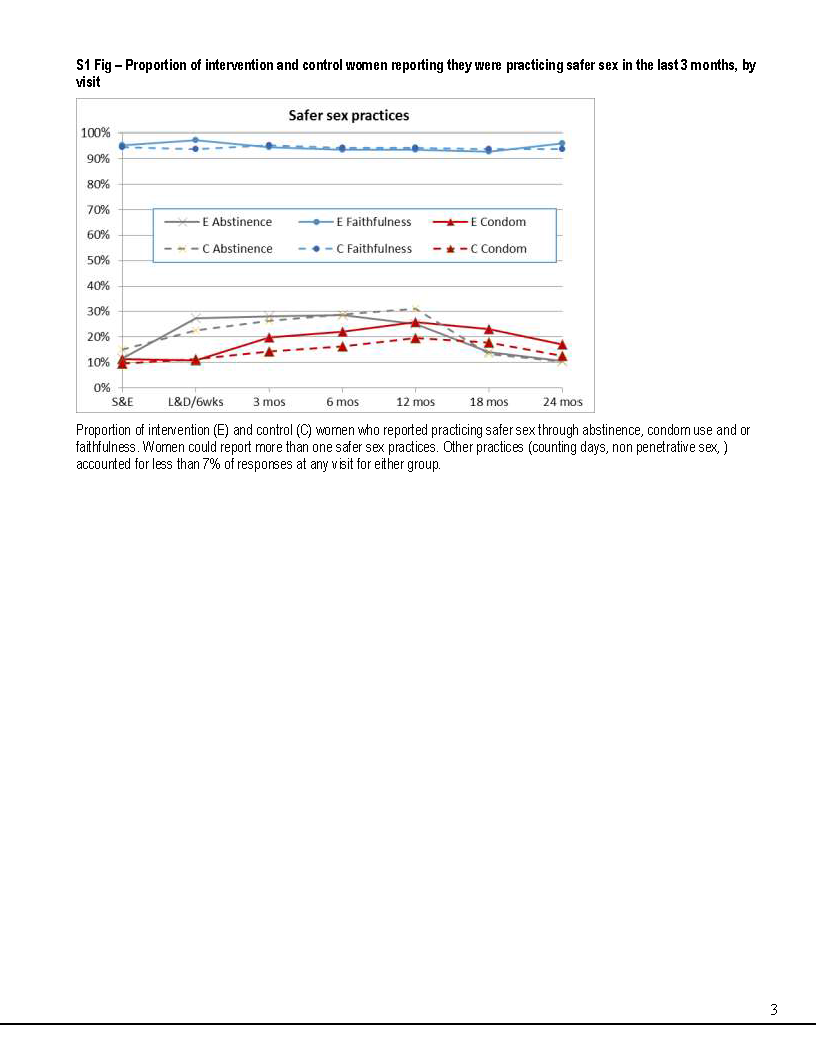

Supplement: S1 Fig — Proportion of intervention (E) and control (C) women who reported practicing safer sex through abstinence, condom use and or faithfulness. Women could report more than one safer sex practices. Other practices (counting days, non-penetrative sex) accounted for less than 7% of responses for either group at any visit. (TIF) [file pone.0212119.s006.tif]

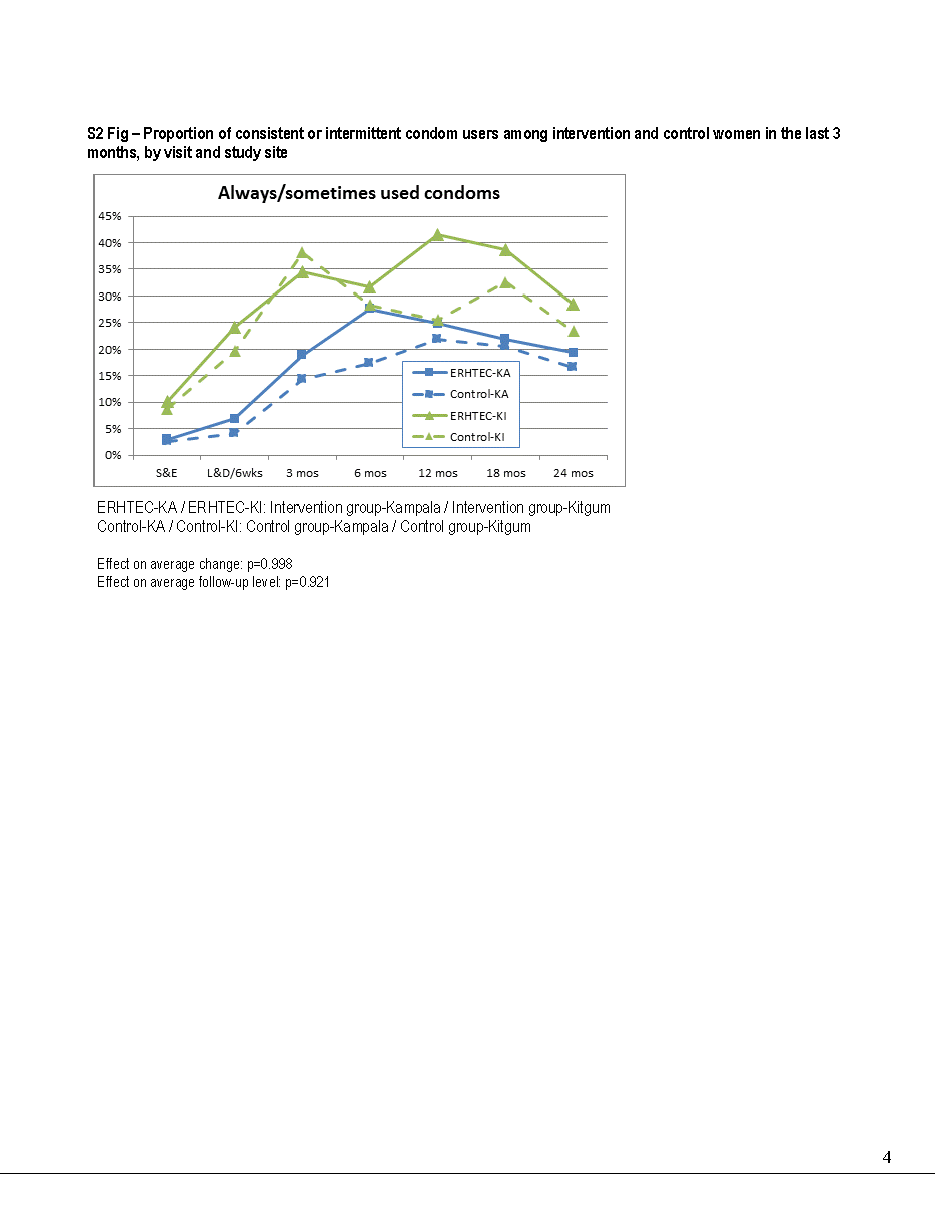

Supplement: S2 Fig — ERHTEC-KA / ERHTEC-KI: Intervention group-Kampala / Intervention group-Kitgum. Control-KA / Control-KI: Control group-Kampala / Control group-Kitgum. Effect on average change: p = 0.998. Effect on average follow-up level: p = 0.921. (TIF) [file pone.0212119.s007.tif]

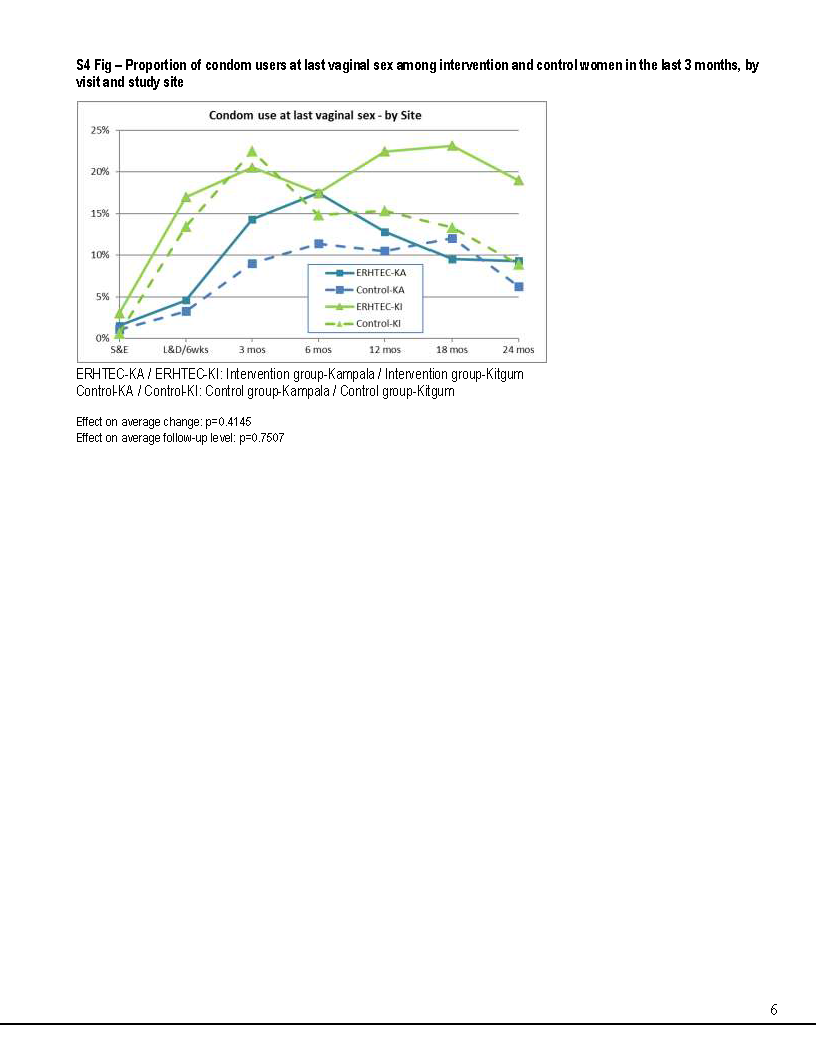

Supplement: S4 Fig — ERHTEC-KA / ERHTEC-KI: Intervention group-Kampala / Intervention group-Kitgum. Control-KA / Control-KI: Control group-Kampala / Control group-Kitgum. Effect on average change: p = 0.415. Effect on average follow-up level: p = 0.751. (TIF) [file pone.0212119.s009.tif]

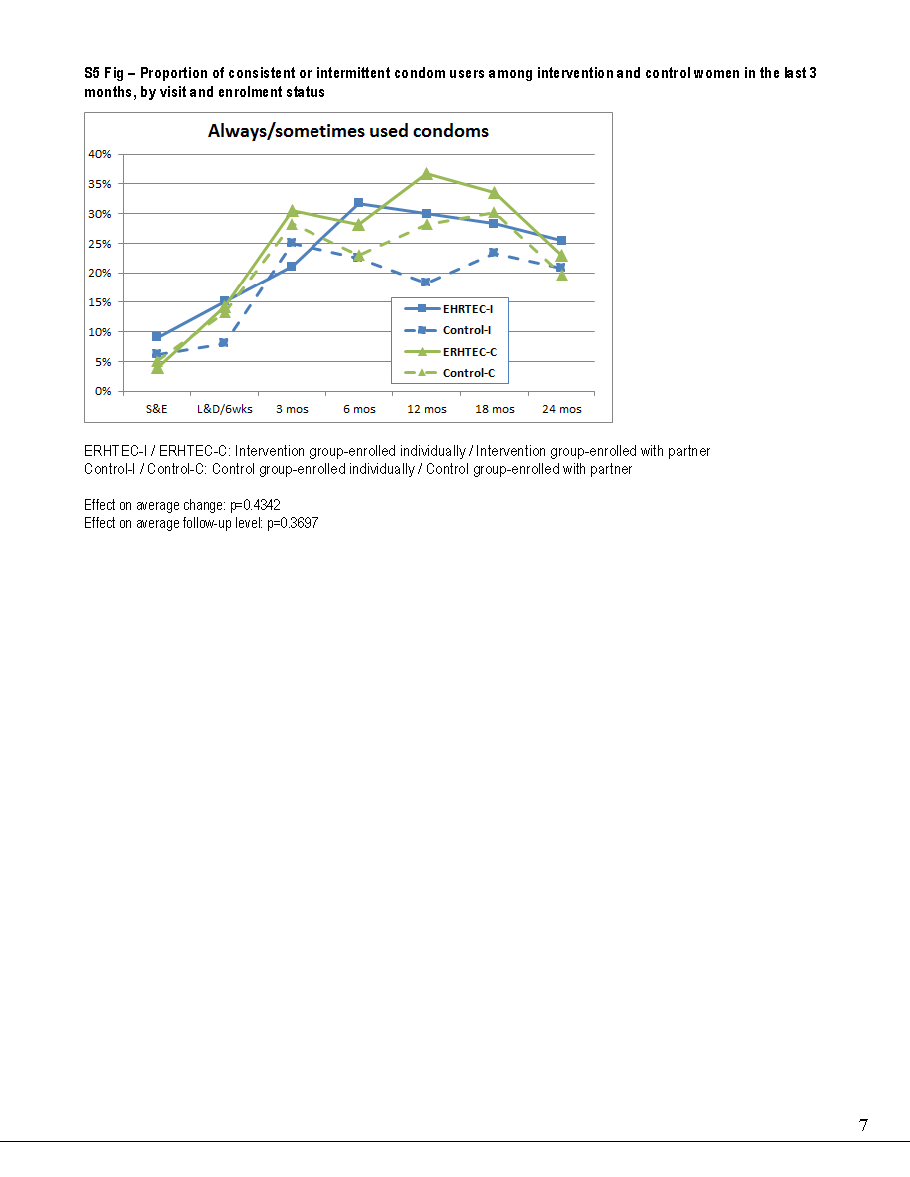

Supplement: S5 Fig — ERHTEC-I: Intervention group-enrolled individually. ERHTEC-C: Intervention group-enrolled with partner. Control-I: Control group-enrolled individually. Control-C: Control group-enrolled with partner. Effect on average change: p = 0.434. Effect on average follow-up level: p = 0.370. (TIF) [file pone.0212119.s010.tif]

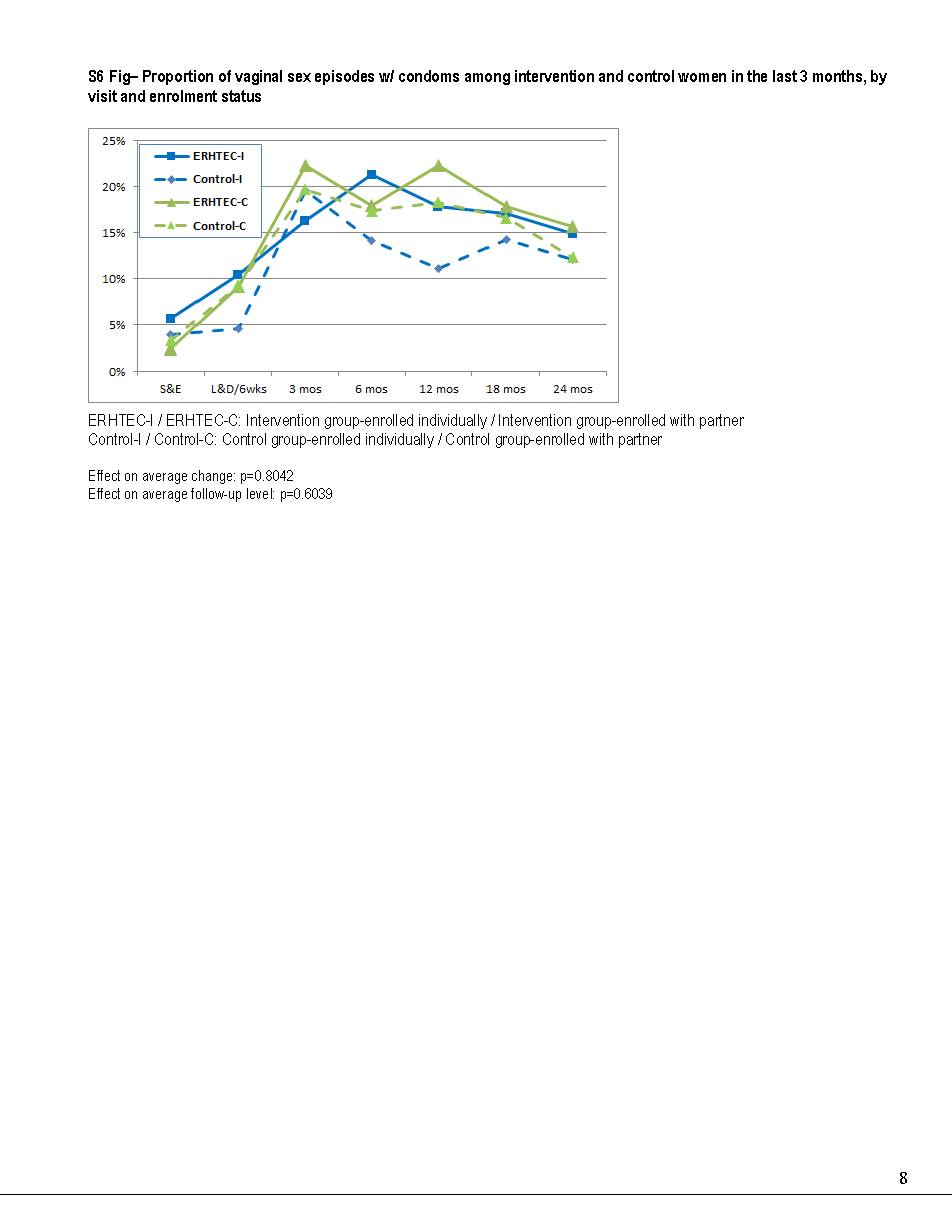

Supplement: S6 Fig — ERHTEC-I: Intervention group-enrolled individually. ERHTEC-C: Intervention group-enrolled with partner. Control-I: Control group-enrolled individually. Control-C: Control group-enrolled with partner. Effect on average change: p = 0.804. Effect on average follow-up level: p = 0.604. (TIF) [file pone.0212119.s011.tif]

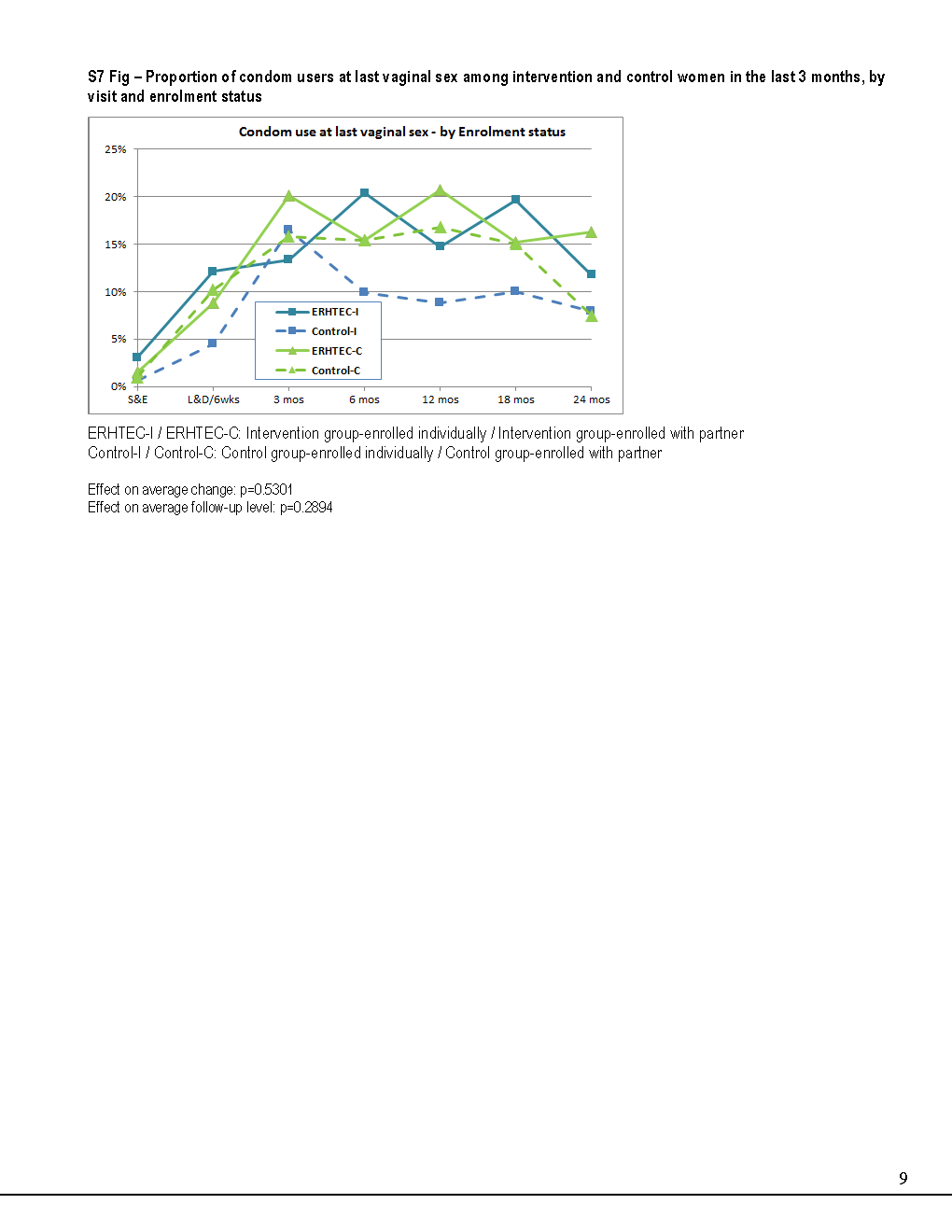

Supplement: S7 Fig — ERHTEC-I: Intervention group-enrolled individually. ERHTEC-C: Intervention group-enrolled with partner. Control-I: Control group-enrolled individually. Control-C: Control group-enrolled with partner. Effect on average change: p = 0.530. Effect on average follow-up level: p = 0.290. (TIF) [file pone.0212119.s012.tif]
