# Supplementary material for: Primary HIV prevention in pregnant and lactating Ugandan women: A randomized trial
Source: PLoS One. 2019 Feb 25;14(2):e0212119. doi: 10.1371/journal.pone.0212119 (PMC6388930; doi:10.1371/journal.pone.0212119)
Supplement: S3 Fig — ERHTEC-KA / ERHTEC-KI: Intervention group-Kampala / Intervention group-Kitgum. Control-KA / Control-KI: Control group-Kampala / Control group-Kitgum. Effect on average change: p = 0.648. Effect on average follow-up level: p = 0.693. (PDF) [file pone.0212119.s008.pdf]

S3 Fig – Proportion of vaginal sex episodes with condoms among intervention and control women in the last 3 months, by visit and study site

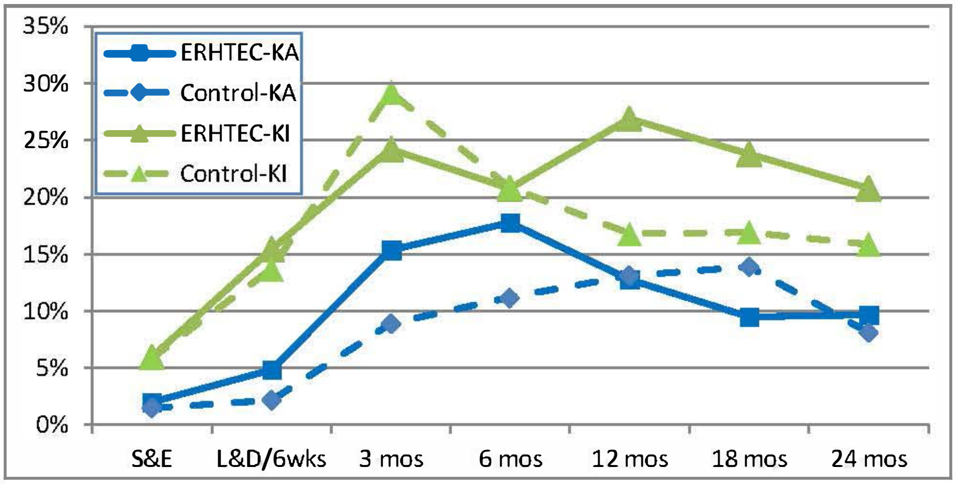

ERHTEC-KA / ERHTEC-KI: Intervention group-Kampala / Intervention group-Kitgum
Control-KA / Control-KI: Control group-Kampala / Control group-Kitgum

Effect on average change: p=0.648
Effect on average follow-up level: p=0.693
